# TP53-based interaction analysis identifies cis-eQTL variants for TP53BP2, FBXO28, and FAM53A that associate with survival and treatment outcome in breast cancer

**DOI:** 10.18632/oncotarget.15110

**Published:** 2017-02-05

**Authors:** Rainer Fagerholm, Sofia Khan, Marjanka K. Schmidt, Montserrat GarcClosas, Päivi Heikkilä, Jani Saarela, Jonathan Beesley, Maral Jamshidi, Kristiina Aittomäki, Jianjun Liu, H. Raza Ali, Irene L. Andrulis, Matthias W. Beckmann, Sabine Behrens, Fiona M. Blows, Hermann Brenner, Jenny Chang-Claude, Fergus J. Couch, Kamila Czene, Peter A. Fasching, Jonine Figueroa, Giuseppe Floris, Gord Glendon, Qi Guo, Per Hall, Emily Hallberg, Ute Hamann, Bernd Holleczek, Maartje J. Hooning, John L. Hopper, Agnes Jager, Maria Kabisch, kConFab/AOCS Investigators, Renske Keeman, Veli-Matti Kosma, Diether Lambrechts, Annika Lindblom, Arto Mannermaa, Sara Margolin, Elena Provenzano, Mitul Shah, Melissa C. Southey, Joe Dennis, Michael Lush, Kyriaki Michailidou, Qin Wang, Manjeet K. Bolla, Alison M. Dunning, Douglas F. Easton, Paul D.P . Pharoah, Georgia Chenevix-Trench, Carl Blomqvist, Heli Nevanlinna

**Affiliations:** ^1^ Department of Obstetrics and Gynecology, Helsinki University Hospital, University of Helsinki, Helsinki, Finland; ^2^ Netherlands Cancer Institute, Antoni van Leeuwenhoek Hospital, Amsterdam, The Netherlands; ^3^ Division of Cancer Epidemiology and Genetics, National Cancer Institute, Rockville, MD, USA; ^4^ Department of Pathology, Helsinki University Hospital, University of Helsinki, Helsinki, Finland; ^5^ Institute for Molecular Medicine Finland (FIMM), University of Helsinki, Finland; ^6^ Department of Genetics, QIMR Berghofer Medical Research Institute, Brisbane, Australia; ^7^ Department of Clinical Genetics, Helsinki University Hospital, University of Helsinki, Helsinki, Finland; ^8^ Human Genetics Division, Genome Institute of Singapore, Singapore, Singapore; ^9^ Cancer Research UK Cambridge Institute, University of Cambridge, Li Ka Shing Centre, Cambridge, UK; ^10^ Department of Pathology, University of Cambridge, Cambridge, UK; ^11^ Lunenfeld-Tanenbaum Research Institute of Mount Sinai Hospital, Toronto, Canada; ^12^ Department of Molecular Genetics, University of Toronto, Toronto, Canada; ^13^ Department of Gynaecology and Obstetrics, University Hospital Erlangen, Friedrich-Alexander University Erlangen-Nuremberg, Comprehensive Cancer Center Erlangen-EMN, Erlangen, Germany; ^14^ Division of Cancer Epidemiology, German Cancer Research Center (DKFZ), Heidelberg, Germany; ^15^ Centre for Cancer Genetic Epidemiology, Department of Oncology, University of Cambridge, Cambridge, UK; ^16^ Division of Clinical Epidemiology and Aging Research, German Cancer Research Center (DKFZ), Heidelberg, Germany; ^17^ German Cancer Consortium (DKTK), German Cancer Research Center (DKFZ), Heidelberg, Germany; ^18^ Division of Preventive Oncology, German Cancer Research Center (DKFZ) and National Center for Tumor Diseases (NCT), Heidelberg, Germany; ^19^ University Cancer Center Hamburg (UCCH), University Medical Center Hamburg-Eppendorf, Hamburg, Germany; ^20^ Department of Laboratory Medicine and Pathology, Mayo Clinic, Rochester, MN, USA; ^21^ Department of Medical Epidemiology and Biostatistics, Karolinska Institutet, Stockholm, Sweden; ^22^ David Geffen School of Medicine, Department of Medicine Division of Hematology and Oncology, University of California at Los Angeles, Los Angeles, CA, USA; ^23^ Usher Institute of Population Health Sciences and Informatics, The University of Edinburgh Medical School, Edinburgh, UK; ^24^ Leuven Multidisciplinary Breast Center, Department of Oncology, KULeuven, Leuven Cancer Institute, University Hospitals Leuven; ^25^ Department of Health Sciences Research, Mayo Clinic, Rochester, MN, USA; ^26^ Molecular Genetics of Breast Cancer, German Cancer Research Center (DKFZ), Heidelberg, Germany; ^27^ Saarland Cancer Registry, Saarbrcken, Germany; ^28^ Department of Medical Oncology, Family Cancer Clinic, Erasmus MC Cancer Institute, Rotterdam, The Netherlands; ^29^ Centre for Epidemiology and Biostatistics, Melbourne School of Population and Global health, The University of Melbourne, Melbourne, Australia; ^30^ Peter MacCallum Cancer Center, The University of Melbourne, Melbourne, Australia; ^31^ Cancer Center of Eastern Finland, University of Eastern Finland, Kuopio, Finland; ^32^ Institute of Clinical Medicine, Pathology and Forensic Medicine, University of Eastern Finland, Kuopio, Finland; ^33^ Imaging Center, Department of Clinical Pathology, Kuopio University Hospital, Kuopio, Finland; ^34^ Vesalius Research Center, VIB, Leuven, Belgium; ^35^ Laboratory for Translational Genetics, Department of Oncology, University of Leuven, Leuven, Belgium; ^36^ Department of Molecular Medicine and Surgery, Karolinska Institutet, Stockholm, Sweden; ^37^ Department of Oncology-Pathology, Karolinska Institutet, Stockholm, Sweden; ^38^ Department of Oncology, University of Cambridge, Addenbrookes Hospital, Cambridge, UK; ^39^ Department of Histopathology, Addenbrookes Hospital, Cambridge University Hospitals NHS Foundation Trust, Cambridge, UK; ^40^ Cambridge Experimental Cancer Medicine Centre and NIHR Cambridge Biomedical Research Centre, Cambridge, UK; ^41^ Department of Pathology, The University of Melbourne, Melbourne, Australia; ^42^ Centre for Cancer Genetic Epidemiology, Department of Public Health and Primary Care, University of Cambridge, Cambridge, UK; ^43^ Department of Electron Microscopy/Molecular Pathology, The Cyprus Institute of Neurology and Genetics, Nicosia, Cyprus; ^44^ Department of Oncology, Helsinki University Hospital, University of Helsinki, Helsinki, Finland; ^45^ Department of Oncology, University of Örebro, Örebro, Sweden

**Keywords:** breast cancer, TP53, survival, anthracycline, SNP

## Abstract

TP53 overexpression is indicative of somatic *TP53* mutations and associates with aggressive tumors and poor prognosis in breast cancer. We utilized a two-stage SNP association study to detect variants associated with breast cancer survival in a TP53-dependent manner. Initially, a genome-wide study (*n* = 575 cases) was conducted to discover candidate SNPs for genotyping and validation in the Breast Cancer Association Consortium (BCAC). The SNPs were then tested for interaction with tumor TP53 status (*n* = 4,610) and anthracycline treatment (*n* = 17,828). For SNPs interacting with anthracycline treatment, siRNA knockdown experiments were carried out to validate candidate genes.

In the test for interaction between SNP genotype and TP53 status, we identified one locus, represented by rs10916264 (p_(interaction)_ = 3.44 05E010^-5^; FDR-adjusted *p* = 0.0011) in estrogen receptor (ER) positive cases. The rs10916264 AA genotype associated with worse survival among cases with ER-positive, TP53-positive tumors (hazard ratio [HR] 2.36, 95% confidence interval [C.I] 1.45 - 3.82). This is a cis-eQTL locus for *FBXO28* and *TP53BP2*; expression levels of these genes were associated with patient survival specifically in ER-positive, *TP53*-mutated tumors. Additionally, the SNP rs798755 was associated with survival in interaction with anthracycline treatment (p_(interaction)_ = 9.57 05E010^-5^, FDR-adjusted *p* = 0.0130). RNAi-based depletion of a predicted regulatory target gene, *FAM53A*, indicated that this gene can modulate doxorubicin sensitivity in breast cancer cell lines.

If confirmed in independent data sets, these results may be of clinical relevance in the development of prognostic and predictive marker panels for breast cancer.

## INTRODUCTION

Genetic variation contributes to the phenotype and prognosis of breast cancer, as high-penetrance mutations and common variants correlate with various histopathological features, most notably estrogen receptor status [1]. The prognosis and indicated treatment for breast cancer is influenced by tumor grade, stage, HER2 expression, and hormone receptor status, and it is plausible that genetic variants associated with these features would be of prognostic and predictive interest. Additionally, genetic variation may contribute to breast cancer survival independently of these markers, potentially by affecting the efficacy of the treatment. For example, prognostic and predictive SNPs have been discovered in the *TP53* gene and its regulatory network, as well as in genes involved in oxidative stress [2–6].

TP53 is a key tumor suppressor involved in several cellular stress response pathways that regulate the cell cycle, apoptosis, senescence, and DNA repair. Somatic *TP53* mutations are common in most types of cancer, including breast cancer where *TP53* mutations have been estimated to occur in 20-30% of cases [7–9]. These mutations are most commonly dominant-negative missense mutations; truncating loss of function mutations are seen in less than 5% of breast cancers [10–12]. The dominant-negative missense mutations lead to the accumulation of mutated *TP53* protein in cell nuclei, which is generally detectable by immunohistochemistry, although it must be noted that the concordance between immunohistochemical detection and sequencing is less than 75% when accounting for truncating mutations and missense mutations outside the conserved regions of the protein [12, 13].

Mutated *TP53*, detected either by immunohistochemistry or by sequencing, has been reported to associate strongly with aggressive tumor phenotypes, e.g. estrogen receptor negativity and high grade, and poor breast cancer survival [2, 7, 8, 10, 11, 14]. The prognostic value of *TP53* mutation status appears to be particularly strong in ER-positive cases, however [11]. Furthermore, *TP53* mutations may also influence disease outcome depending on the type of treatment, at least in the case of endocrine therapy for ER-positive cancer [15]. *TP53* mutations have also been suggested to modulate sensitivity to anthracycline-cyclophosphamide combination chemotherapy, possibly in interaction with ER status, although the clinical significance of these findings remains inconclusive [12, 16, 17, 18]. We hypothesize that genetic variants that influence TP53-related biological processes may have a TP53-dependent association with breast cancer survival. Such effects could be masked by *TP53* mutations or occur exclusively in *TP53*-mutated cancers. To test this hypothesis, we have utilized a two-stage study design to search for genetic variants associated with survival in TP53-related breast cancer.

## RESULTS

### Rs10916264 and TP53 IHC have an interactive association with survival in ER-positive cases

First, we performed an initial genome-wide screen in the HEBCS-GWS data set (N = 572) for candidate SNPs that may be associated with survival in a TP53-dependent manner. In this analysis, 111 SNPs met our selection criteria and were also represented on the BCAC iCOGS array and therefore selected for further validation (Supplementary Table 1). In total, 136 BCAC SNPs matched the 111 HEBCS-GWS candidate SNPs at a LD threshold of r^2^ > 0.8.

Next, we performed an interaction analysis between SNP genotypes and TP53 immunohistochemistry (IHC) status in the BCAC validation series (N = 4610) (Table 1). At this stage, five closely linked SNPs emerged as statistically significant, the strongest being rs10916264 (p_(interaction)_ = 3.44 × 10^-5^; p = 0.0011 after Benjamini-Hochberg adjustment) among ER-positive tumors. These SNPs are in high LD with each other (minimum r^2^ 0.93, D’ 0.97) and therefore represent the same association signal. This interaction remained statistically significant after adjustment for standard prognostic factors (tumor size, grade, and node status). The Cox proportional hazards models for the rs10916264:TP53 interaction in ER-positive cases are presented in Table 2. The hazard ratio of the interaction term 2.06 (1.47 – 2.91) is consistent with the corresponding HEBCS-GWS SNP rs6604887 (r^2^ 0.93, D’ 0.97) despite the low power in the ER-positive HEBCS-GWS subset (HR 1.94, 95% C.I. 0.82 – 4.02). In a comparison of the Cox models in ER-positive and ER-negative BCAC cases, the interaction terms suggested opposite effects and this difference was statistically significant (p = 0.0022, z-test for heterogeneity). The same was seen in the HEBCS-GWS data set, even though the interactions themselves did not reach statistical significance in either ER-based subgroup (heterogeneity p = 0.04).

**Table 1:** Description of the data sets used in this study

	HEBCS GWS	BCAC P53-based data set	BCAC chemotherapy data set
No. of cases	805	4610	17828
Vital status			
Alive	466 (58%)	3847 (83%)	15630 (88%)
Deceased: all-cause	339 (42%)	763 (17%)	2198 (12%)
Follow-up mean ±SD (years)	10.6 ± 6.6	8.5 ± 4.3	7.3 ± 4.0
Age at diagnosis, mean [range]	54.1 [22 – 87]	54.4 [20 – 95]	55.2 [19 – 95]
ER			
Negative	230 (29%)	925 (20%)	3002 (17%)
Positive	513 (64%)	3473 (75%)	11753 (66%)
Missing data	62 (8%)	212 (5%)	3073 (17%)
Grade			
1	144 (18%)	931 (20%)	2911 (16%)
2	312 (39%)	2004 (43%)	6354 (36%)
3	280 (35%)	1323 (29%)	4414 (25%)
Missing data	69 (9%)	352 (8%)	4149 (23%)
Tumor size category ^a^			
1	390 (48%)	2663 (58%)	9338 (52%)
2	304 (38%)	1438 (31%)	4615 (26%)
3	50 (6%)	105 (2%)	635 (4%)
4	47 (6%)	-	-
Missing data	14 (2%)	404 (9%)	3240 (18%)
N (nodal metastasis)			
Negative	338 (42%)	2552 (55%)	8976 (50%)
Positive	446 (55%)	1649 (36%)	5471 (31%)
Missing data	21 (3%)	409 (9%)	3381 (19%)
P53 immunohistochemistry			
Negative	418 (52%)	3824 (83%)	4204 (24%)
Positive	157 (20%)	786 (17%)	976 (5%)
Missing data	230 (29%)	0 (0%)	12648 (71%)
Adjuvant chemotherapy treatment			
No adjuvant chemotherapy	445 (55%)	2472 (54%)	11108 (62%)
Anthracycline+Taxane	14 (2%)	79 (2%)	733 (4%)
Anthracycline ^b^	191 (24%)	397 (9%)	2277 (13%)
Taxane	2 (0.2%)	17 (0.4%)	135 (0.8%)
Methotrexate ^b^	153 (19%)	222 (5%)	1022 (6%)
Unknown regimen	-	697 (15%)	2528 (14%)
Missing data	-	734 (16%)	-
Adjuvant endocrine treatment			
Treated	282 (35%)	2769 (60%)	11340 (64%)
Not treated	520 (65%)	1506 (33%)	5670 (32%)
Missing data	3 (0.4%)	335 (7%)	818 (5%)

^a^ T-stage for HEBCS-GWS. For BCAC, categorized based on tumor size: 1 = < 20 mm, 2 = 20-50 mm, 3 = >50 mm.

^b^ In BCAC, approximately 5% of methotrexate-treated cases also received anthracycline treatment, and 2% of anthracycline-treated cases also received methotrexate.

**Table 2:** Proportional hazards models depicting the interaction between rs10916264 genotype (additive model) and TP53 immunohistochemistry in ER-positive cases, and between rs798755 genotype (recessive model) and adjuvant anthracycline chemotherapy

	rs10916264:TP53 IHC ^a^				rs798755:Anthracycline ^b^		
Model without interaction term	HR (95% C.I.)		p-value		HR (95% C.I.)		p-value
SNP	0.93 (0.82 – 1.06)		0.22914		0.95 (0.74 – 1.22)		0.6970
TP53 IHC	1.56 (1.23 – 2.00)		0.00033		-		-
Anthracycline treatment	-		-		1.68 (1.47 – 1.92)		1.5 × 10^-14^
Age at diagnosis	1.05 (1.04 – 1.06)		< 10^-16^		1.04 (1.04 – 1.05)		< 10^-16^
							
Model with interaction term	HR (95% C.I.)		p-value		HR (95% C.I.)		p-value
SNP	0.83 (0.73 – 0.96)		0.0093		0.72 (0.52 – 0.98)		0.0352
TP53 IHC	0.83 (0.54 – 1.25)		0.3602		-		-
Anthracycline treatment	-		-		1.61 (1.40 – 1.84)		6.9 × 10^-12^
Age at diagnosis	1.05 (1.04 – 1.06)		< 10^-16^		1.04 (1.04 – 1.05)		< 10^-16^
SNP:TP53 interaction	2.06 (1.47 – 2.91)		3.3 × 10^-5^		-		-
SNP:Anthracycline interaction	-		-		2.99 (1.78 – 5.01)		3.5 × 10^-5^
							
Likelihood-ratio test for interaction	p_(interaction)_ = 3.44 × 10^-5^				p_(interaction)_ = 9.57 × 10^-5^		
							
Adjusted for prognostic factors	HR (95% C.I.)		p-value		HR (95% C.I.)		p-value
SNP	0.87 (0.75 – 1.01)		0.0712		0.92 (0.63 – 1.36)		0.6883
TP53 IHC	0.77 (0.49 – 1.23)		0.2748		-		-
Anthracycline treatment	-		-		0.98 (0.80 – 1.20)		0.8487
Age at diagnosis	1.06 (1.05 – 1.07)		< 10^-16^		1.05 (1.04 – 1.06)		< 10^-16^
Estrogen receptor (ER) status	-		-		0.73 (0.61 – 0.86)		2.5 × 10^-5^
Grade	1.39 (1.19 – 1.62)		3.6 × 10^-5^		1.40 (1.26 – 1.55)		5.3 × 10^-10^
Tumor size (mm)	1.01 (1.01 – 1.02)		3.2 × 10^-5^		1.01 (1.01 – 1.02)		1.6 × 10^-14^
Node status	1.78 (1.44 – 2.19)		7.2 × 10^-8^		1.88 (1.63 – 2.17)		< 10^-16^
SNP:TP53 interaction	1.69 (1.16 – 2.46)		0.0047		-		-
SNP:Anthracycline interaction	-		-		2.30 (1.08 – 4.86)		0.0299
							

^a^ Additive model, among ER-positive cases only

^b^ Recessive model, all cases

**Figure 1:**
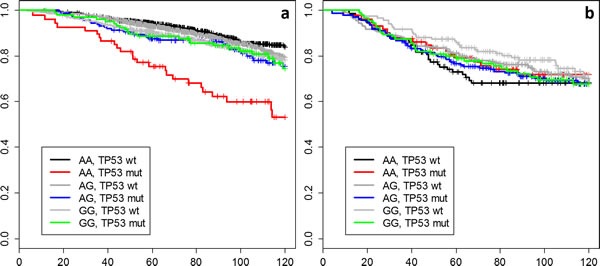
Kaplan-Meier curves for all combinations of rs10916264 genotype and TP53 status among pooled **a**) ER-positive and **b**) ER-negative BCAC cases.

**Figure 2:**
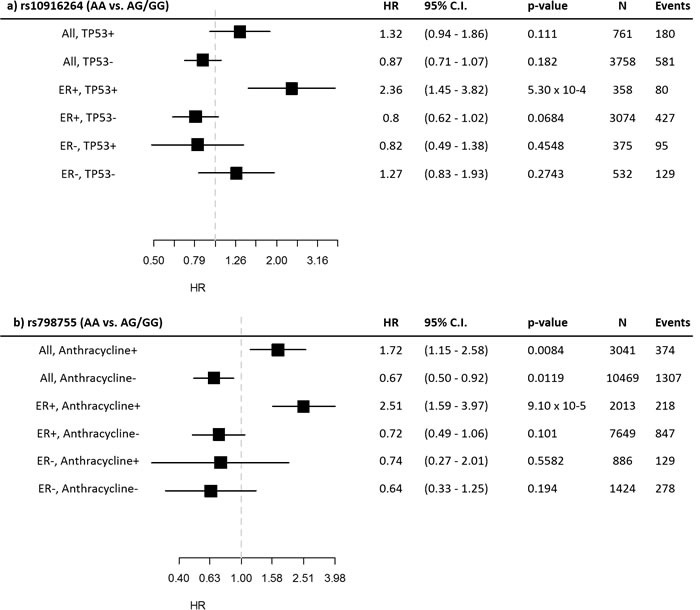
Subgroup statistics of the SNPs detected in the interaction analyses Hazard ratios and confidence intervals are displayed for the recessive model in the indicated subgroups for a) rs10916264 and b) rs798755.

**Figure 3:**
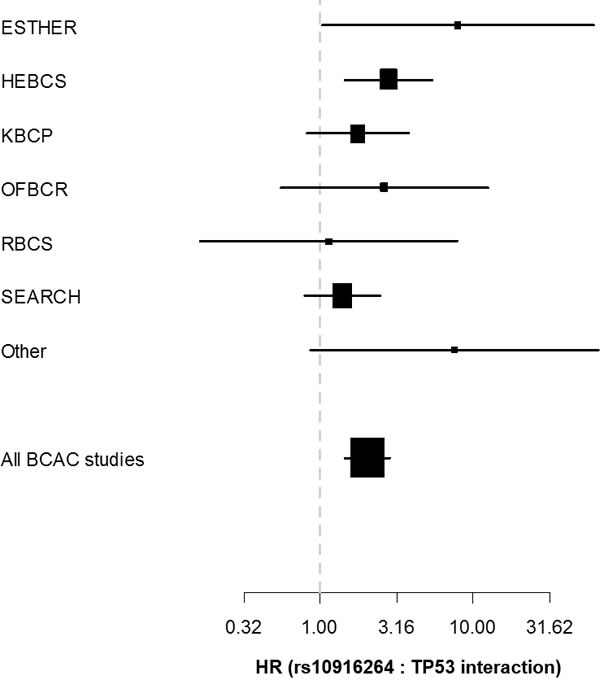
Forest plot depicting the hazard ratio (x-axis) and corresponding confidence intervals for the rs10916264:TP53 interaction term (additive genetic model among ER-positive cases) separately in each eligible BCAC study Studies with fewer th were pooled into the “Other” category.

To better illustrate the interaction results, we plotted Kaplan-Meier curves of all genotype-IHC combinations (Figure 1). The plots indicate that specifically the rs10916264 AA ancestral genotype (genotype frequency 23.2%, allele frequency 48.0%; used as reference allele in the interaction analysis) associates with worse survival among ER-positive, TP53-positive cases, while all other genotype-IHC combinations are clustered together. We therefore also analyzed this SNP using the recessive genetic model, using G as the reference allele, and calculated Cox proportional hazard models in TP53- and ER-based subgroups. In ER-positive, TP53-positive breast cancer cases, the homozygous rs10916264 AA genotype associated with a HR of 2.36 (95% confidence interval [C.I.] 1.45 – 3.82; Figure 2a), while in ER-positive, TP53-negative cases, the homozygous genotype did not associate with a difference in survival at all (HR 0.80, 95% C.I. 0.62 – 1.02; Figure 2a). When the rs10916264:TP53 interaction model was calculated separately for each study, all studies were in agreement on the direction of the interaction term (I^2^ = 11.72%, Q test for heterogeneity p = 0.3927; Figure 3). The interaction remained statistically significant (p = 2.6 × 10-4) when adjuvant endocrine therapy was included in the Cox model, and the interaction exists at a nominally significant level even among ER-positive cases not treated with endocrine therapy (n = 843, 140 events, p = 0.04). The interaction was also independent of adjuvant chemotherapy treatment with nominally significant interaction p-values in both chemotherapy-treated and untreated groups (p = 0.033 and 0.028, respectively).

No statistically significant SNP:P53 interactions were detected in the full BCAC data set (when ER-status was ignored), nor in ER-negative cases. A complete listing of all interaction test p-values can be found in Supplementary Table 2. See also Supplementary Table 3 for a list of all candidate SNPs from the HEBCS-GWS pilot, the corresponding BCAC SNPs, and linkage disequilibrium statistics between the two.

### Rs798755 associates with survival after anthracycline therapy

As positive TP53 IHC correlates with aggressive tumor characteristics that often indicate adjuvant chemotherapy treatment in the clinic, some SNPs observed to interact with TP53 IHC in our pilot study may reflect response to treatment rather than a true biological interaction with TP53. We therefore also conducted a treatment-based interaction analysis irrespective of TP53 status, restricting the interaction test to anthracycline-based regimens as this was the commonly used treatment type in both HEBCS-GWS and BCAC data sets. One statistically significant SNP emerged under the recessive genetic model: rs798755 (p_(interaction)_ = 9.57 × 10^-5^; p = 0.0130 after Benjamini-Hochberg adjustment) (Supplementary Table 2). The Cox proportional hazards models describing this interaction are presented in Table 2. Upon further analysis of genotype-specific hazards, the rs798755 homozygous AA-genotype (genotype frequency 4.0%, allele frequency 19.7%) was associated with an increased hazard in anthracycline-treated cases (HR 1.72, 95% C.I. 1.15 – 2.58), and a decreased hazard in cases not treated with anthracyclines (HR 0.67, 95% C.I. 0.50 – 0.92; includes also cases that received no adjuvant chemotherapy). In the HEBCS-GWS data set, the corresponding SNP rs798766 (r^2^ 1.0, D’ 1.0) also associated with an increased hazard in anthracycline-treated cases (HR 2.62, 95% C.I. 1.30 – 5.29), although we saw no evidence of a protective effect in untreated cases (HR 1.54, 95% C.I. 0.88 – 2.72).

An analysis restricted to BCAC cases receiving non-anthracycline chemotherapy (instead of all cases not treated with anthracyclines) suffers from low statistical power, but the HR estimate is similar to the above (HR 0.65, 95% C.I. 0.29 – 1.47). The interaction between rs798755 and anthracycline therapy may be dependent on ER status: an increased hazard was seen in ER-positive, anthracycline-treated cases (HR 2.51, 95% C.I. 1.59 – 3.97), but not in ER-negative, anthracycline-treated cases (HR 0.74, 95% C.I. 0.27 – 2.01). (Figure 2b)

### Characterization of the rs10916264 locus

The rs10916264 locus is located at 1q42.11 in a promoter-flanking regulatory region (Ensembl regulatory region ID ENSR00001772409) between the genes *TP53BP2* and *FBXO28*. The maximum LD region (r^2^ > 0.1) around the SNP contains the genes *CAPN8*, *TP53BP2*, *FBXO28*, *DEGS1*, *CNIH4*, and *WDR26*. Computational annotation of correlated SNPs using multiple sources of genomic data revealed several potentially functional variants. Intersection of variants with genomic features relevant to target gene prediction methods suggested several potential target genes. For example, SNPs correlated (at r2>0.8) with rs10916264 overlap regulatory marks associated with CHIA-PET signals that interact with the promoters of *CNIH4* and *FBXO28*. Enhancers associated with expression of *FBXO28* and *DEGS1* also harbor highly correlated SNPs. Many of these variants overlap annotated regulatory features (such as Roadmap and ENCODE enhancers, promoters and transcription factor binding sites) and consequently exhibit RegulomeDB scores suggestive of functional impact. These results are presented in detail in Supplementary Table 4.

Our eQTL analyses of METABRIC gene expression data indicate that rs10916264 and its tagging SNPs associate with the expression of *FBXO28* also in breast tumor tissue (p = 0.0016). The rs10916264 A-allele was associated with higher expression of *FBXO28*. We also noted two additional *cis*-eQTLs between the rs10916264 locus and the genes *TP53BP2* (p = 0.0087) and *CNIH3* (p = 0.0023). Of these, *FBXO28* and *CNIH3* were predicted to be regulatory targets of the variants in this region. No statistically significant *trans*-eQTLs were detected.

Analysis of Kaplan-Meier Plotter data indicates that high *FBXO28* expression is associated with poor survival among ER-positive breast cancer cases (HR 1.57, 95% C.I. 1.35 – 1.81; p = 9.5 × 10^-10^). Similar to the rs10916264 SNP, this effect is not seen in ER-negative cases: the calculated HR for high *FBXO28* expression would in fact suggest a protective effect (HR 0.81, 95% C.I: 0.63 – 1.03) although the difference is not statistically significant (p = 0.081). Restricting the analysis to cases with known sequence-based *TP53* mutation status greatly reduces statistical power, but the association between *FBXO28* and survival is consistent with the SNPs in the rs10916264 locus. High *FBXO28* expression was associated with poor survival in ER-positive, TP53-mutated cases (HR 2.35, 95% C.I. 1.17 – 4.72, p = 0.0133), but not in ER-positive TP53 wild-type cases (HR 1.27, 95% C.I. 0.82 – 1.96, p = 0.2886). A similar result was seen for *TP53BP2*, where high expression had a protective effect in ER-positive, *TP53*-mutated cases (HR 0.27, 95% C.I. 0.12 – 0.63, p = 0.001), but not in ER-positive TP53-wild type cases (HR 0.82, 95% C.I. 0.53 – 1.27, p = 0.383) or in ER-negative cases (HR 1.19, 95% C.I. 0.91 – 1.56, p = 0.191). The direction of the hazard ratios for *TP53BP2* and *FBXO28* were consistent with the directions of the eQTLs observed for the survival-associated SNPs in the region. Unlike *FBXO28* and *TP53BP2*, the expression level of *CNIH3* did not associate with survival.

### Characterization of the rs798755 locus

The rs798755 LD region (r^2^ > 0.1) on chromosome 4 contains the genes *FAM53A*, *SLBP*, *TMEM129*, and *TACC3*, all of which are in eQTL association with the SNPs in the region in numerous cell types, although no such correlation has been reported specifically in breast tissue. The SNP is located in a regulatory region (ENSR00002001253) containing multiple active promoter and enhancer histone marks in a variety of tissues including breast. At this locus, SNPs correlated with rs798755 (r^2^ > 0.8) overlap with multiple MCF7 ChIA-PET signals that interact with the promoters of the above four genes as well as a number of other genes further along the chromosome: *FGFR3, MXD4, WHSC1*, RP11-572O17.1 and *NELFA*. Correlated SNPs also intersect with computationally predicted enhancers linked to expression of *TACC3* and *SLBP*. Many highly correlated variants coincide with regulatory elements in normal and tumor breast cell types, including enhancers, promoters and transcription factor binding sites. In our METABRIC eQTL analysis of breast tumor data, SNPs correlated with rs798755 were associated with the expression of *TACC3* (p = 0.00099). See Supplementary Table 4 for details on the predicted regulatory sites in this region. Gene expression based survival analyses were not feasible for these genes, since data on specific adjuvant chemotherapy regimens was not available in the Kaplan-Meier Plotter database.

### siRNA knockdown of FAM53A expression influences doxorubicin sensitivity in breast cancer cells

**Figure 4:**
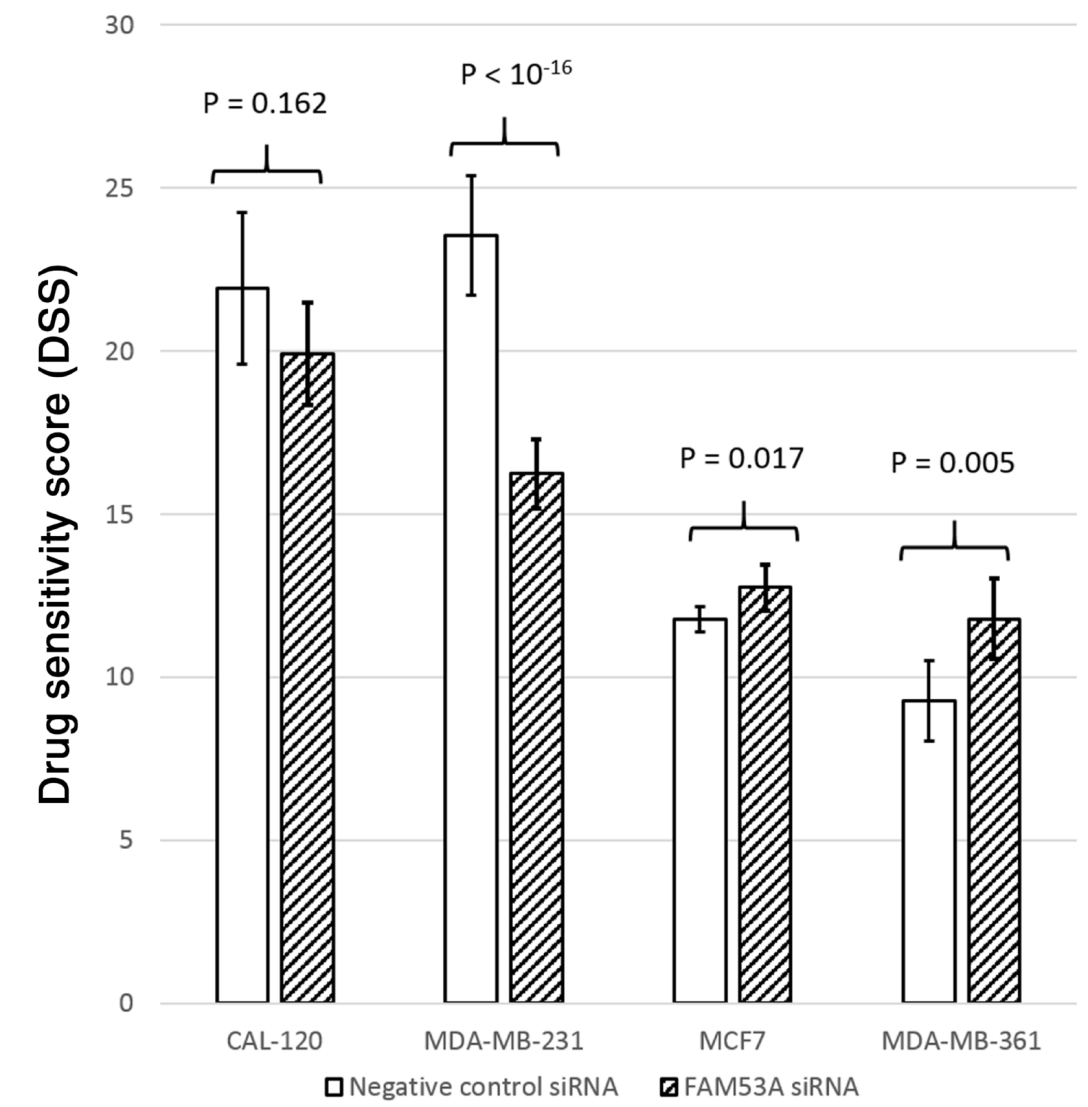
Drug Sensitivity Scores (DSS) for cells transfected with *FAM53A* siRNAs and negative control siRNAs in each of the four cell lines Higher DSS scores indicate better sensitivity to doxorubicin.

To test whether the expression levels of the genes surrounding rs798755 influence anthracycline response in breast cancer cell lines (CAL-120, MCF7, MDA-MB-231, and MDA-MB-361), we tested whether siRNA knockdown of these genes influences the doxorubicin dose response of the cells. Of the genes in the rs798755 region, *FAM53A* knockdown resulted in increased doxorubicin resistance (lower DSS) compared to the negative control siRNA in the triple-negative, TP53-mutated MDA-MB-231 cells (p < 10^-16^). In CAL-120 cells, a similar but statistically non-significant effect was seen between FAM53A siRNAs and negative controls (p = 0.162). In the Luminal-B MDA-MB-361 and Luminal-A MCF7 cell lines, *FAM53A* knockdown instead resulted in increased sensitivity to doxorubicin (p = 0.005 and p = 0.017, respectively). See Figure 4 for a visual comparison of DSS scores between *FAM53A* siRNAs and negative controls in the four cell lines. Knockdown of *SLBP*, *TMEM129*, or *TACC3* did not influence doxorubicin sensitivity in any of the cell lines.

## DISCUSSION

**Figure 5:**
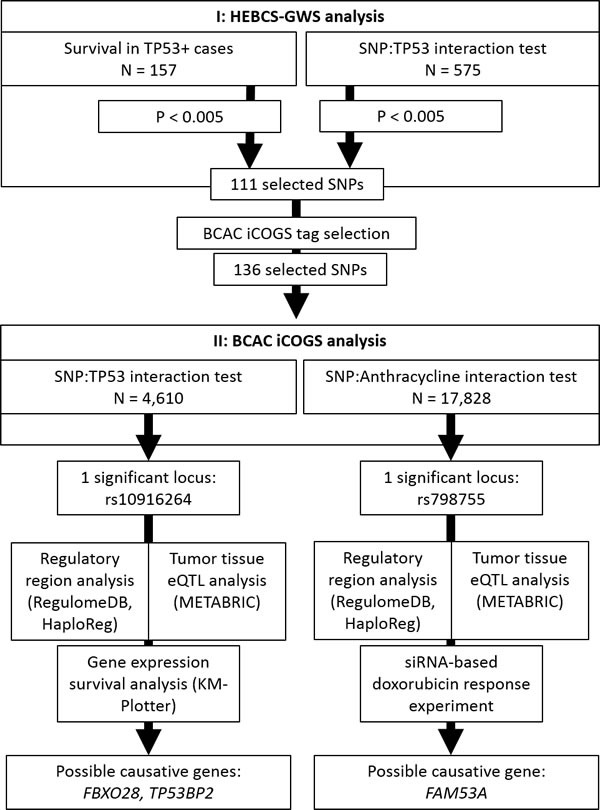
Diagram of the workflow of this study

We have performed a two stage SNP association study for the purpose of discovering genetic variants that may influence breast cancer survival in a TP53-dependent manner. The general workflow of the study is illustrated in Figure 5. In an interaction test between SNP genotypes and TP53 overexpression, one locus (represented by rs10916264 and linked SNPs) emerged as statistically significant among ER-positive cases, independently of conventional prognostic factors; this effect differed significantly from ER-negative cases. Although initially detected using the additive genetic model, the effect appears to be recessive: specifically the rs10916264 AA-genotype is associated with poor survival in TP53- and ER-positive cases. The survival difference in this group of patients is remarkable: rs10916264 appears to distinguish a subgroup of TP53-positive cases with poor prognosis among ER-positive cases (Figure 1a). No association of rs10916264 with survival was seen in the ER-positive, TP53-negative cases, nor in ER-negative cases. The interaction was not seen in the main analysis of all BCAC cases, but this is consistent with the effect direction differing between ER-positive and ER-negative cases. This effect appeared to be independent of both endocrine and adjuvant chemotherapy treatment, although more detailed analyses of specific chemotherapy regimens were not carried out due to lack of statistical power.

The rs10916264 locus is in a promoter-flanking region upstream of the *FBXO28* gene, a predicted target of the regulatory variants in LD with rs10916264. The rs10916264 A-allele correlates with increased expression of *FBXO28*, which in turn was associated with poor breast cancer survival in the Kaplan-Meier Plotter database. Similar to the SNP, *FBXO28* expression was only associated with survival in ER-positive breast cancers with somatic *TP53* mutations. A similar but opposite effect was seen for *TP53BP2*, also consistent with the eQTL direction: low *TP53BP2* expression associated with adverse prognosis in ER-positive cases with *TP53* mutations. This lends credence to the idea that genetic variation at this locus influences breast cancer survival through regulation of one or both of the genes *TP53BP2* and *FBXO28*.

Our evidence points to *FBXO28* as the strongest candidate gene in the region: it is predicted to be a target of the regulatory variants, its expression in breast tumor tissue correlates with these variants, and its expression level also associates with breast cancer survival specifically in ER-positive, TP53-mutated breast cancer. Functional FBXO28 activity has also been shown to be associated with adverse breast cancer prognosis, and correlates with *TP53* mutation status specifically in ER-positive breast tumors [19, 20]. FBXO28 belongs to the F-box family of proteins that determine the substrate specificity of the SCF ubiquitin ligase complex, a regulatory system that plays a critical role in tumorigenesis [21, 22]. SCF-FBXO28 specifically ubiquitylates Myc, promoting Myc-p300 transcriptional activity and subsequent oncogenic signaling [19]. Increased *FBXO28* gene expression alone is not sufficient for this process: FBXO28 activation requires phosphorylation by the cyclin-dependent kinases CDK1 and CDK2. Since CDK activity is regulated by TP53 through p21 [23], this provides another rationale for why increased FBXO28 expression would influence survival predominantly in TP53-mutated cancer.

While *TP53BP2* was not a predicted target in our computational analysis, variants in strong LD with rs10916264 did associate with its expression in breast tumors. This, along with the gene's well-characterized interaction with TP53, makes it also a strong candidate to functionally connect the regulatory variants in this region to breast cancer survival. The association between low *TP53BP2* expression and poor survival in *TP53*-mutated breast cancer seems counterintuitive at first, because TP53BP2 binds TP53 to induce apoptosis, and this pro-apoptotic cooperation can be defective or absent when TP53 is mutated [24, 25]. However, TP53BP2 also has TP53-independent binding partners and tumor suppressor activities that can partially compensate for defective TP53. TP53BP2 can bind IκB and induce repression of p63 through NF-κB, suppressing tumorigenesis and metastasis in squamous cell carcinoma [26]. It can also inhibit autophagy by binding Ras [27], a mechanism through which TP53BP2 has been shown to enhance oxaliplatin-induced apoptosis in colorectal cancer cells independently of TP53 [28]. Furthermore, TP53BP2 can regulate proliferation and apoptosis in the *TP53*-mutated MDA-MB-231 breast cancer cell line [29].

Certain caution is advisable in the interpretation of these results, since TP53 immunohistochemistry as used in this study is not a comprehensive method for *TP53* mutation detection. The detectable over-abundance of TP53 results from typically dominant-negative *TP53* missense mutations that lead to the accumulation of a stable but dysfunctional form of the protein. The study material therefore includes an unknown proportion of cases with other types of somatic mutations; the concordance between TP53 immunohistochemistry and DNA sequencing has previously been estimated to be below 75%, mainly due to truncating mutations [12, 13]. The immunohistochemically detectable dominant-negative mutations may be of particular interest, however, as they have been reported to confer oncogenic activity to TP53 [30]. It is also of note that the initial SNP selection in this study relies on a small sample set with relatively little statistical power, which may have led to missed SNPs as well as an inflated number of false positives in the initial candidate SNP set. Our goal was to offset this by the increase in statistical power achieved by focusing the validation analysis on a fairly small set of SNPs, resulting in a lesser degree of multiple testing and therefore more power to detect weak to moderate effect sizes (HRs). Given that positive TP53 status was strongly associated with aggressive tumor characteristics and therefore correlated strongly with the administration of adjuvant chemotherapy, we speculated that the initial TP53-based signal in HEBCS-GWS might in fact reflect an interaction with treatment. When we tested for interaction between candidate SNP genotypes and anthracycline treatment, the SNP rs798755 emerged as statistically significant. This locus (rs798766, r^2^ = 1) has previously been shown to associate with urinary bladder cancer risk and recurrence [31]. Our breast tumor eQTL and target prediction analyses pointed to *TACC3* as the gene most likely affected by variants in this region. Overexpression of *TACC3* has previously been associated with oncogenic activity, defective DNA repair, and poor survival in breast and lung cancer [32]. This would be consistent with the association of the rs798755 minor allele with high *TACC3* expression in our eQTL analysis of breast tumor data. However, *TACC3* siRNA knockdown did not influence doxorubicin sensitivity in the breast cancer cell lines we tested, although we cannot rule out the possibility that this gene may contribute to the observed association with survival *in vivo*.

Previously published studies have identified rs798755 as a cis-eQTL locus for the *FAM53A* gene in a wide variety of tissue types. *FAM53A* belongs to a vertebrate-specific family of three homologous genes of largely unknown function: *FAM53A, FAM53B*, and *FAM53C* [33]. In the siRNA experiment, *FAM53A* knockdown affected doxorubicin response significantly in three out of four tested cell lines. The *TP53*-mutated, basal MDA-MB-231 cells were rendered more doxorubicin-resistant by *FAM53A* depletion, and a similar but statistically non-significant effect was seen in the similarly basal and *TP53*-mutated CAL-120 cell line, while the luminal MDA-MB-361 (truncating *TP53* mutation) and MCF7 (*TP53* wild-type) cells became more sensitive to doxorubicin. It is tempting to speculate that this effect is caused by the basal-luminal difference, and/or the presence of gain-of-function *TP53* mutations in the two basal cell lines, but more cell lines would have to be examined to support such conclusions, and further study is required to elucidate the underlying biological mechanisms and their *in vivo* relevance. These findings are consistent with our SNP-based clinical findings and eQTL data, however: the rs798755 A-allele associates with poor prognosis in ER-positive, anthracycline-treated cases, as well as with higher *FAM53A* expression, which based on our siRNA experiments correlates with anthracycline resistance in ER-positive cell lines. It can therefore be speculated that FAM53A may play a role in the commonly seen resistance to anthracyclines and other chemotherapy drugs in ER-positive breast cancer [34–36].

The functions of FAM53A and its paralogues are not well understood. The best-known member of the family is FAM53B, a controller of tissue development and cell proliferation [33, 37]. FAM53B is required for Wnt signaling, a pathway involved in epithelial-to-mesenchymal transition and subsequent metastasis in breast cancer [38–40]. *FAM53B* has also been identified as a critical gene in the prognosis of multiple myeloma in a transcriptional network analysis [41]. FAM53B has also been shown to bind 14-3-3 chaperones, a family of proteins known to play a key role in cellular resistance to anticancer drugs, including doxorubicin [33, 42–44]. *FAM53A* is therefore a plausible candidate to influence anthracycline response in breast cancer, even though the mechanism of action cannot be speculated on in any detail, and the results concerning rs798755 and its associated genes must be considered hypothesis-generating without immediate clinical relevance. If confirmed, however, these results may aid researchers in understanding the mechanisms of anthracycline resistance in ER-positive breast cancer, which in turn can facilitate clinical research towards improved individualized therapy.

In conclusion, we have identified a regulatory genetic locus in 1q42.11, represented by rs10916264 and other SNPs in its LD region, which distinguishes a group with poor survival after breast cancer specifically in cases with TP53 overabundance in ER-positive tumors. This genetic variation may influence the expression and/or regulation of several genes in the region, with the evidence pointing most strongly to *FBXO28* and *TP53BP2*. This result may provide useful clues to the relationship between TP53 and ER signaling in breast cancer as well as to the way this interplay influences tumor progression and the outcome of the disease. We have also identified a *cis*-eQTL variant for *FAM53A* that may associate with response to anthracycline treatment in ER-positive breast cancer. Both of these genetic loci may provide useful prognostic and/or predictive genetic markers that, if validated, may be of clinical use in identifying cases likely to benefit from specific treatments or more aggressive treatment regimens. Detailed investigation of the biological and clinical significance of these variants requires further study.

## MATERIALS AND METHODS

### Discovery GWS (HEBCS-GWS)

The collection and genotyping of the HEBCS-GWS series has been previously described [45]. In total, genotype information was obtained from a study series consisting of 805 Finnish breast cancer cases (HEBCS-GWS), enriched for cases with distant metastasis or death at the time of the initiation of the study in 2008: the series includes 312 breast cancer specific events, and 339 any-cause mortality events. TP53 immunohistochemistry data was available for 575 cases [2]; these cases comprise the discovery series. All cases were female, ascertained for their first primary invasive breast cancer. See Table 1 for a detailed description of this data set.

### Validation (BCAC)

Candidate SNPs identified in the HEBCS GWAS analysis were included on a custom Illumina Infinium array (iCOGS) for large-scale genotyping of a data set of 50,927 individuals from 52 Breast Cancer Association Consortium (BCAC) member studies [46]. Each of the host institutions of the respective study recruited under ethically approved protocols by the local institutional review boards. BCAC studies represented on the iCOGS chip were included in survival analysis if sufficient follow-up data was available, with a minimum requirement of at least ten survival events (deaths from any cause) per study. Additionally, we included only studies with cases from predominantly European ancestry (in total 99.7% of individuals known to be of European origin), and that provided data on either adjuvant treatment or TP53 immunohistochemistry. See Supplementary Table 5 for a description of these studies. Clinicopathological data was collected as previously described [47], including TP53 immunohistochemistry data from 3,476 cases. Tumor tissue microarray (TMA) samples from an additional set of 1,134 individual cases (from five BCAC studies) were stained for TP53 and scored centrally at the Helsinki University Hospital using monoclonal mouse anti-human DO-7 antibody (Dako Inc., Carpinteria, CA 93013, USA) at a 1:200 dilution. Slides were pre-treated for 60 minutes in CC1 buffer (Ventana Inc., Tucson, AZ 85755, USA) and stained using the Ventana Benchmark XT system (Ventana, USA). The Ultraview Universal DAB Detection kit 760-500 (Ventana, USA) was used for detection. Scoring was categorized into a positive/negative score based on a cutoff of >20 % clearly positive tumor nuclei. See Supplementary Table 6 for a listing of the TP53 staining and scoring methods for each study.

Two partially overlapping subsets of the BCAC data set were used. For the primary TP53 interaction analysis, we selected all cases with available genotype, TP53 immunohistochemistry, and survival data (N = 4,610). For an analysis of chemotherapy-related effects, we included all cases with available survival and adjuvant chemotherapy information, irrespective of TP53 data availability (N = 17,828). These data sets are described in detail in Table 1.

### Statistical analysis

Survival statistics were calculated using Cox proportional hazards models. The preliminary HEBCS-GWS survival analysis was carried out using two different endpoints in parallel: five-year BDDM (breast cancer death or distant metastasis), and 10-year overall survival (death from any cause). Two different approaches were used: (i) survival analyses restricted to TP53-positive cases only (N = 157), and (ii) interaction analyses between SNP genotypes and TP53 immunohistochemistry in all cases (N = 575). Only the log-additive genetic model was analyzed at this stage. SNPs associating with survival at p < 0.005 in either of the two tests were selected as candidates for validation in the next stage. If the SNP itself was not present on the iCOGS genotyping chip, tagging SNPs were selected using 1000genomes r^2^ > 0.8 as the minimum LD threshold for proxy SNP selection.

At the validation stage (BCAC), 10-year overall survival (death from any cause) was used as the end point in all survival analyses. Follow-up times were left-truncated to account for case recruitment latency. All Cox models were adjusted for age at diagnosis and stratified by study. The primary analysis consisted of an interaction test between SNP genotypes and TP53 immunohistochemistry using the log-additive genetic model; statistical significance was determined by a likelihood ratio test. The following groups were analyzed: all cases, ER-positive cases, and ER-negative cases. For statistically significant SNPs, we also calculated multivariate Cox interaction models that included the following standard prognostic factors in addition to age: estrogen receptor status (ER), histological grade, nodal metastasis (N), and tumor size.

In the anthracycline treatment based analyses, interaction terms of the form SNP*Anthracycline were introduced to the Cox models. To take advantage of the greater statistical power in this data set, both the additive and recessive genetic models were analyzed. Statistically significant hits from the BCAC interaction analysis were tested for consistency of effect direction in HEBCS-GWS within identically defined subgroups. Taxane- and methotrexate-based regimens were not investigated, as these regimens are less representative of the original HEBCS-GWS material, and the numbers were deemed to be too small for an adequately powered interaction analysis. Benjamini-Hochberg correction was used to confirm statistical significance in the presence of multiple testing [48].

### eQTL analysis

To determine if the survival-associated SNPs or other SNPs in the LD region (r^2^ > 0.1) associate with gene expression in breast cancer, we utilized the publicly available METABRIC data set [49]. The METABRIC gene expression data was generated by the Illumina Human WG6 v3 platform. Tumor tissue genotyping had been carried out using the Affymetrix Genome Wide Human SNP array 6.0. METABRIC consists of 1,328 breast tumor samples with both genotype and gene expression data. eQTL analysis was carried out by calculating linear models between genotype and gene expression using the R package *‘MatrixEQTL’* [50]. We searched for *cis*-eQTLs within regions defined as ±1 Mb from any SNP in the LD region. Any genes outside these regions were analyzed for *trans*-eQTL and subjected to transcriptome-wide multiple testing correction (Benjamini-Hochberg).

### Further *in silico* evaluation of SNPs and candidate genes

It is likely that the prognostic SNPs identified in this study are merely linkage disequilibrium proxies for other, functionally significant variants in their genomic vicinity. In an effort to identify the SNPs and genes with a direct functional impact on breast cancer survival, we utilized a number of public databases. For these analyses, regions of interest were defined as regions containing SNPs in any linkage disequilibrium (LD) with the survival-associated SNPs at r^2^> 0.1. Linked SNPs in these regions were analyzed for their impact on regulatory features using a target gene prediction pipeline that utilizes publicly available chromosome conformation capture, promoter prediction, super-enhancer, and enhancer promoter cap data to connect regulatory variants to likely target genes in breast-derived cell types as previously described [51]. These data were intersected with the breast cancer specific eQTL results from METABRIC to identify the most likely target genes. For a detailed listing of the target prediction resources, see Supplementary Table 4. Candidate genes in the regions surrounding the prognostic SNPs were analyzed in *Kaplan-Meier plotter*, a gene expression and survival database [52]. These analyses were performed using 10-year relapse-free survival, as this was the most widely available end-point, and optimized break points for the binarization of gene expression levels.

### siRNA transfection / drug response measurement of breast cancer cell lines

In the case of SNPs that associated with survival in anthracycline-treated cases, we performed a siRNA knockdown based drug response experiment to test whether the genes in the LD regions surrounding the SNPs influence doxorubicin response in breast cancer cell lines. The target genes and selected siRNAs were *FAM53A*, *SLBP*, *TACC3*, and *TMEM129*. Three different siRNAs were used per gene (see Supplementary Table 7 for details), and assayed separately, each with five replicates. The cell lines used were MCF7 (luminal A, TP53 wild-type), MDA-MB-361 (luminal B, TP53 truncating mutation), MDA-MB-231 (triple-negative, TP53 missense mutation), and CAL-120 (triple-negative, TP53 missense mutation). Cells were transfected with siRNAs and 24h later supplemented with five different doxorubicin concentrations: 0.83, 10, 100, 500, and 1000 nM. The number of viable cells was measured after 71h doxorubicin treatment. Based on cell viability at increasing doxorubicin concentrations, drug response curves were calculated and converted into Drug Sensitivity Score (DSS) statistics [53]. DSS scores for each target gene were then pooled across replicates and siRNAs and compared to cells transfected with a target-less negative control siRNA in twelve replicates using Student's t-test.

### Data availability

The relevant BCAC SNP genotype data underpinning these analyses can be accessed by applying to the BCAC consortium (http://bcac.ccge.medschl.cam. ac.uk/). The dataset can be made available by the BCAC coordinating centre upon request to the corresponding authors and with the permission of BCAC Data Access Coordination Committee.

## SUPPLEMENTARY MATERIALS TABLES















## Abbreviations

BCAC: Breast Cancer Association Consortium
SNP: Single Nucleotide Polymorphism
ER: Estrogen Receptor
eQTL: Expression Quantitative trait Loci
HR: Hazard Ratio
C.I.: Confidence Interval
FDR: False Discovery Rate
GWS: Genome-Wide association Study
IHC: Immunohistochemistry
siRNA: Small Interfering RNA
LD: Linkage Disequilibrium
CDK: Cyclin-Dependent Kinase
TMA: Tumor Tissue Microarray
N: Lymph Node metastasis
