# Supplementary material for: TP53-based interaction analysis identifies cis-eQTL variants for TP53BP2, FBXO28, and FAM53A that associate with survival and treatment outcome in breast cancer
Source: Oncotarget. 2017 Feb 5;8(11):18381–98. doi: 10.18632/oncotarget.15110 (PMC5392336; doi:10.18632/oncotarget.15110)
Supplement: Supplementary file 1 [file oncotarget-08-18381-s001.docx]

**Supplementary Table 1.** Candidate SNPs selected from HEBCS genome-wide survival analysis. Hazard ratios and confidence intervals are presented for overall survival (death from any cause within 10 years) and BDDM (death from breast cancer or distant metastasis within 5 years) in the P53-positive subgroup (N = 157). Additionally, p_(interaction)_ values are presented for an interaction test between SNP genotype and P53 overexpression in the entire data set (N = 575). SNPs with p < 0.005 were selected for the validation analyses in BCAC.

| **Overall survival (10 years)** | | | | | **BDDM (5 years)** | | | | |
| --- | --- | --- | --- | --- | --- | --- | --- | --- | --- |
| **SNP** | **HR** | **95% C.I.** | **p-value** | **p_(interaction)_** | | **HR** | **95% C.I.** | **p-value** | **p_(interaction)_** |
| rs498498 | 0.49 | 0.35 - 0.68 | 2.56E-05 | 9.01E-07 | | 0.50 | 0.36 - 0.68 | 1.5E-05 | 1.8228E-06 |
| rs653920 | 0.49 | 0.35 - 0.68 | 2.56E-05 | 9.01E-07 | | 0.50 | 0.36 - 0.68 | 1.5E-05 | 1.8228E-06 |
| rs9553874 | 0.50 | 0.36 - 0.7 | 4.80E-05 | 1.62E-06 | | 0.50 | 0.37 - 0.69 | 2.6E-05 | 2.83419E-06 |
| rs4769504 | 1.99 | 1.43 - 2.75 | 4.04E-05 | 3.69E-06 | | 2.02 | 1.47 - 2.78 | 1.67E-05 | 1.83795E-06 |
| rs9553864 | 0.50 | 0.36 - 0.7 | 4.04E-05 | 3.84E-06 | | 0.49 | 0.36 - 0.68 | 1.67E-05 | 3.27668E-06 |
| rs7986966 | 1.78 | 1.3 - 2.42 | 0.000267 | 5.06E-05 | | 1.82 | 1.35 - 2.46 | 9.34E-05 | 6.00428E-06 |
| rs1018123 | 0.56 | 0.41 - 0.77 | 0.000267 | 5.06E-05 | | 0.55 | 0.41 - 0.74 | 9.34E-05 | 6.00428E-06 |
| rs9604787 | 0.49 | 0.32 - 0.75 | 0.000971 | 0.004001492 | | 0.41 | 0.28 - 0.61 | 1.13E-05 | 0.000630566 |
| rs221417 | 0.28 | 0.16 - 0.5 | 1.83E-05 | 0.000437176 | | 0.35 | 0.21 - 0.6 | 0.000109 | 0.01874181 |
| rs2543578 | 0.28 | 0.16 - 0.5 | 1.83E-05 | 0.000437176 | | 0.35 | 0.21 - 0.6 | 0.000109 | 0.01874181 |
| rs606410 | 0.35 | 0.22 - 0.57 | 2.00E-05 | 2.69E-05 | | 0.43 | 0.26 - 0.69 | 0.000516 | 0.003820499 |
| rs2276881 | 0.28 | 0.14 - 0.54 | 0.000187 | 0.015462523 | | 0.24 | 0.13 - 0.47 | 2.07E-05 | 0.001120581 |
| rs7702447 | 0.48 | 0.33 - 0.68 | 5.85E-05 | 4.35E-05 | | 0.48 | 0.34 - 0.68 | 4E-05 | 0.000113992 |
| rs12484656 | 0.51 | 0.36 - 0.71 | 6.47E-05 | 5.23E-05 | | 0.52 | 0.37 - 0.72 | 9.02E-05 | 0.000300785 |
| rs4941434 | 0.46 | 0.31 - 0.7 | 0.000263 | 0.007006263 | | 0.44 | 0.29 - 0.67 | 0.00011 | 0.002254805 |
| rs970694 | 0.61 | 0.45 - 0.82 | 0.001181 | 0.019095667 | | 0.57 | 0.42 - 0.75 | 0.000111 | 0.009928643 |
| rs11155550 | 0.63 | 0.44 - 0.9 | 0.012077 | 0.005408885 | | 0.57 | 0.4 - 0.81 | 0.001542 | 0.000113368 |
| rs7628838 | 1.75 | 1.28 - 2.38 | 0.000463 | 0.008675252 | | 1.79 | 1.33 - 2.41 | 0.000131 | 0.004002478 |
| rs7155894 | 0.52 | 0.37 - 0.73 | 0.000131 | 0.000300476 | | 0.55 | 0.4 - 0.77 | 0.000459 | 0.005652733 |
| rs179734 | 1.93 | 1.38 - 2.71 | 0.000131 | 0.000388469 | | 1.81 | 1.3 - 2.53 | 0.000459 | 0.006879272 |
| rs575381 | 0.41 | 0.26 - 0.65 | 0.000158 | 0.111939591 | | 0.46 | 0.29 - 0.72 | 0.000724 | 0.314165144 |
| rs11711870 | 0.44 | 0.29 - 0.68 | 0.000189 | 0.024673404 | | 0.50 | 0.33 - 0.76 | 0.001106 | 0.101467825 |
| rs7760914 | 2.47 | 1.53 - 3.99 | 0.000227 | 0.00038529 | | 2.25 | 1.43 - 3.54 | 0.000464 | 0.001705905 |
| rs6792584 | 1.72 | 1.25 - 2.35 | 0.000801 | 0.01245871 | | 1.77 | 1.3 - 2.39 | 0.000229 | 0.004400209 |
| rs9941427 | 1.67 | 1.16 - 2.41 | 0.006296 | 0.000260167 | | 1.44 | 1 - 2.06 | 0.048422 | 0.015638589 |
| rs3096337 | 1.82 | 1.3 - 2.56 | 0.00056 | 0.027461449 | | 1.84 | 1.32 - 2.54 | 0.000261 | 0.025346545 |
| rs310834 | 0.49 | 0.34 - 0.72 | 0.000273 | 0.007362697 | | 0.56 | 0.39 - 0.81 | 0.002125 | 0.036150953 |
| rs1546010 | 1.67 | 1.16 - 2.41 | 0.006296 | 0.00028579 | | 1.44 | 1 - 2.06 | 0.048422 | 0.016885211 |
| rs1830115 | 0.30 | 0.16 - 0.58 | 0.000289 | 0.003948269 | | 0.46 | 0.24 - 0.9 | 0.023229 | 0.137911991 |
| rs6018611 | 0.25 | 0.12 - 0.56 | 0.000747 | 0.000297305 | | 0.37 | 0.17 - 0.81 | 0.012499 | 0.045839808 |
| rs4856867 | 0.60 | 0.44 - 0.82 | 0.001213 | 0.014375208 | | 0.58 | 0.43 - 0.78 | 0.000299 | 0.004545957 |
| rs1275993 | 0.51 | 0.33 - 0.8 | 0.003534 | 0.076916441 | | 0.46 | 0.3 - 0.7 | 0.000299 | 0.055369797 |
| rs1885277 | 1.93 | 1.35 - 2.77 | 0.000306 | 0.000871012 | | 1.78 | 1.26 - 2.51 | 0.001046 | 0.023795493 |
| rs7819002 | 1.92 | 1.31 - 2.81 | 0.000861 | 0.000390619 | | 1.51 | 1.01 - 2.25 | 0.04397 | 0.040793009 |
| rs1874786 | 0.57 | 0.42 - 0.78 | 0.00047 | 0.012197841 | | 0.63 | 0.46 - 0.85 | 0.00291 | 0.032632344 |
| rs11822684 | 2.43 | 1.47 - 4 | 0.000494 | 0.005330656 | | 2.00 | 1.21 - 3.29 | 0.006669 | 0.036827315 |
| rs4643731 | 1.77 | 1.28 - 2.44 | 0.000557 | 0.003427631 | | 1.70 | 1.24 - 2.34 | 0.001094 | 0.016653413 |
| rs1133603 | 1.80 | 1.18 - 2.74 | 0.005916 | 0.000578272 | | 1.37 | 0.92 - 2.05 | 0.126052 | 0.168468408 |
| rs1509778 | 1.76 | 1.27 - 2.43 | 0.000652 | 0.003639531 | | 1.71 | 1.24 - 2.34 | 0.000914 | 0.015154282 |
| rs760645 | 1.76 | 1.26 - 2.46 | 0.000947 | 0.013016473 | | 1.76 | 1.27 - 2.43 | 0.000666 | 0.060680102 |
| rs6595079 | 1.73 | 1.26 - 2.38 | 0.000691 | 0.000915771 | | 1.61 | 1.19 - 2.18 | 0.002276 | 0.004946298 |
| rs4344726 | 1.78 | 1.22 - 2.6 | 0.002914 | 0.020027851 | | 1.85 | 1.3 - 2.65 | 0.000705 | 0.034500438 |
| rs1523262 | 0.55 | 0.36 - 0.84 | 0.005916 | 0.000742836 | | 0.73 | 0.49 - 1.09 | 0.126052 | 0.184337837 |
| rs13227862 | 0.50 | 0.33 - 0.75 | 0.000773 | 0.006395244 | | 0.61 | 0.4 - 0.93 | 0.021966 | 0.145463305 |
| rs7739790 | 0.35 | 0.19 - 0.64 | 0.000833 | 0.001853185 | | 0.42 | 0.23 - 0.79 | 0.0066 | 0.06891134 |
| rs350784 | 0.32 | 0.15 - 0.68 | 0.003082 | 0.000864897 | | 0.37 | 0.18 - 0.75 | 0.006018 | 0.002502646 |
| rs12444778 | 0.41 | 0.24 - 0.69 | 0.000874 | 0.007608844 | | 0.45 | 0.27 - 0.75 | 0.002326 | 0.00332958 |
| rs9385608 | 0.55 | 0.39 - 0.78 | 0.000938 | 0.017319708 | | 0.74 | 0.51 - 1.06 | 0.103874 | 0.1395131 |
| rs7605254 | 0.54 | 0.38 - 0.78 | 0.001 | 0.002094306 | | 0.67 | 0.47 - 0.95 | 0.023654 | 0.110477231 |
| rs12533185 | 1.78 | 1.26 - 2.5 | 0.001047 | 0.007528694 | | 1.48 | 1.07 - 2.04 | 0.016291 | 0.134458376 |
| rs17399998 | 0.54 | 0.38 - 0.78 | 0.001054 | 0.02511056 | | 0.60 | 0.41 - 0.87 | 0.007699 | 0.014014296 |
| rs7621006 | 1.83 | 1.28 - 2.63 | 0.00106 | 0.004767333 | | 1.79 | 1.25 - 2.55 | 0.001368 | 0.008179825 |
| rs515028 | 0.25 | 0.11 - 0.57 | 0.001061 | 0.005185238 | | 0.42 | 0.22 - 0.8 | 0.008701 | 0.043560984 |
| rs6907188 | 0.62 | 0.43 - 0.88 | 0.007138 | 0.002479206 | | 0.60 | 0.43 - 0.84 | 0.003393 | 0.001072372 |
| rs2445958 | 1.78 | 1.23 - 2.56 | 0.002027 | 0.022129316 | | 1.81 | 1.27 - 2.59 | 0.001118 | 0.009474485 |
| rs7080287 | 0.49 | 0.31 - 0.76 | 0.001433 | 0.413447533 | | 0.57 | 0.38 - 0.87 | 0.009331 | 0.515937042 |
| rs11920441 | 0.59 | 0.41 - 0.87 | 0.007038 | 0.035932609 | | 0.53 | 0.36 - 0.78 | 0.001215 | 0.001132759 |
| rs10934370 | 1.74 | 1.14 - 2.67 | 0.010168 | 0.001141207 | | 1.33 | 0.88 - 2 | 0.174053 | 0.221608209 |
| rs4720305 | 0.38 | 0.21 - 0.68 | 0.001179 | 0.001337768 | | 0.39 | 0.22 - 0.69 | 0.001174 | 0.005281335 |
| rs1542287 | 0.45 | 0.28 - 0.73 | 0.001181 | 0.007296817 | | 0.47 | 0.29 - 0.75 | 0.001604 | 0.002262827 |
| rs7114163 | 1.41 | 1.01 - 1.97 | 0.046361 | 0.045965489 | | 1.48 | 1.07 - 2.06 | 0.019017 | 0.001203764 |
| rs10481281 | 1.77 | 1.25 - 2.5 | 0.001281 | 0.003414959 | | 1.46 | 1.04 - 2.06 | 0.029265 | 0.07412743 |
| rs11928389 | 1.84 | 1.21 - 2.78 | 0.004018 | 0.013382839 | | 1.95 | 1.29 - 2.95 | 0.001659 | 0.001288173 |
| rs10908367 | 1.65 | 1.13 - 2.42 | 0.009676 | 0.001352785 | | 1.56 | 1.07 - 2.28 | 0.020518 | 0.010910836 |
| rs4377765 | 0.43 | 0.25 - 0.72 | 0.001467 | 0.002049837 | | 0.60 | 0.35 - 1.01 | 0.055168 | 0.018887618 |
| rs17360838 | 1.70 | 1.09 - 2.64 | 0.019226 | 0.037310799 | | 1.83 | 1.23 - 2.73 | 0.003066 | 0.001556869 |
| rs1388970 | 0.57 | 0.41 - 0.81 | 0.001581 | 0.13717745 | | 0.76 | 0.55 - 1.06 | 0.105653 | 0.690845418 |
| rs6604887 | 1.74 | 1.23 - 2.45 | 0.001581 | 0.13717745 | | 1.31 | 0.94 - 1.82 | 0.105653 | 0.690845418 |
| rs2868802 | 2.63 | 1.3 - 5.32 | 0.00712 | 0.001606821 | | 2.01 | 1 - 4.06 | 0.05064 | 0.115878241 |
| rs7746504 | 1.77 | 1.24 - 2.52 | 0.001633 | 0.021935909 | | 1.32 | 0.91 - 1.9 | 0.139367 | 0.166940104 |
| rs11864373 | 1.72 | 1.23 - 2.41 | 0.001654 | 0.09321497 | | 1.63 | 1.18 - 2.27 | 0.00344 | 0.058645994 |
| rs950590 | 0.63 | 0.43 - 0.94 | 0.021936 | 0.025231784 | | 0.56 | 0.39 - 0.8 | 0.00175 | 0.04405354 |
| rs10210979 | 0.39 | 0.22 - 0.7 | 0.001755 | 0.053318192 | | 0.48 | 0.27 - 0.87 | 0.014727 | 0.362565771 |
| rs776021 | 0.54 | 0.36 - 0.79 | 0.001771 | 0.001797183 | | 0.61 | 0.42 - 0.89 | 0.00955 | 0.008180829 |
| rs750358 | 1.72 | 1.17 - 2.52 | 0.006149 | 0.001791869 | | 1.53 | 1.04 - 2.24 | 0.029758 | 0.00418247 |
| rs6018564 | 0.39 | 0.19 - 0.79 | 0.008471 | 0.001816807 | | 0.51 | 0.25 - 1.02 | 0.056663 | 0.123810716 |
| rs2284322 | 0.39 | 0.19 - 0.79 | 0.008471 | 0.001816807 | | 0.51 | 0.25 - 1.02 | 0.056663 | 0.123810716 |
| rs12618367 | 0.39 | 0.21 - 0.7 | 0.00191 | 0.089386499 | | 0.57 | 0.3 - 1.08 | 0.086757 | 0.536970079 |
| rs13099918 | 0.63 | 0.46 - 0.88 | 0.006787 | 0.001928595 | | 0.67 | 0.48 - 0.92 | 0.013838 | 0.120939677 |
| rs2673521 | 1.63 | 1.16 - 2.3 | 0.004879 | 0.103929873 | | 1.71 | 1.22 - 2.39 | 0.001957 | 0.006264472 |
| rs12494912 | 1.52 | 1.11 - 2.09 | 0.009959 | 0.001971608 | | 1.46 | 1.08 - 1.99 | 0.014542 | 0.003880122 |
| rs6886725 | 1.62 | 1.15 - 2.28 | 0.005932 | 0.006532205 | | 1.70 | 1.21 - 2.38 | 0.002225 | 0.003374953 |
| rs2237172 | 1.63 | 1.19 - 2.23 | 0.002433 | 0.002655669 | | 1.59 | 1.17 - 2.16 | 0.003033 | 0.008796593 |
| rs9925768 | 1.68 | 1.2 - 2.35 | 0.002511 | 0.118380947 | | 1.59 | 1.15 - 2.21 | 0.005254 | 0.075454338 |
| rs10876347 | 1.73 | 1.21 - 2.48 | 0.002553 | 0.007555919 | | 1.70 | 1.19 - 2.41 | 0.003192 | 0.057373516 |
| rs2634734 | 0.61 | 0.44 - 0.86 | 0.005239 | 0.039970577 | | 0.60 | 0.44 - 0.84 | 0.002633 | 0.016079647 |
| rs12693085 | 1.76 | 1.22 - 2.54 | 0.002683 | 0.012564124 | | 1.41 | 0.96 - 2.06 | 0.078973 | 0.057431438 |
| rs12078739 | 1.78 | 1.22 - 2.59 | 0.002794 | 0.055177186 | | 1.51 | 1.02 - 2.23 | 0.038298 | 0.192084418 |
| rs1452138 | 1.68 | 1.19 - 2.36 | 0.00285 | 0.049024067 | | 1.49 | 1.07 - 2.07 | 0.017945 | 0.112409727 |
| rs3824954 | 0.60 | 0.42 - 0.84 | 0.00285 | 0.049024067 | | 0.67 | 0.48 - 0.93 | 0.017945 | 0.112409727 |
| rs10764990 | 1.65 | 1.19 - 2.31 | 0.003071 | 0.002999214 | | 1.42 | 1.04 - 1.96 | 0.029441 | 0.009278196 |
| rs6018492 | 2.51 | 1.29 - 4.92 | 0.0071 | 0.003025815 | | 1.98 | 1.02 - 3.88 | 0.044704 | 0.162668488 |
| rs603682 | 1.67 | 1.13 - 2.46 | 0.009356 | 0.003132961 | | 1.51 | 1.05 - 2.18 | 0.026881 | 0.04931217 |
| rs2570069 | 2.23 | 1.31 - 3.8 | 0.003221 | 0.026914238 | | 1.98 | 1.19 - 3.3 | 0.008803 | 0.027263384 |
| rs745052 | 1.61 | 1.16 - 2.23 | 0.004377 | 0.029868341 | | 1.60 | 1.17 - 2.19 | 0.003364 | 0.007527179 |
| rs4919741 | 0.62 | 0.43 - 0.88 | 0.008001 | 0.003472598 | | 0.73 | 0.52 - 1.04 | 0.08357 | 0.008984331 |
| rs11672071 | 1.62 | 1.17 - 2.24 | 0.003598 | 0.016921639 | | 1.42 | 1.04 - 1.94 | 0.029238 | 0.050138783 |
| rs12613687 | 0.60 | 0.43 - 0.85 | 0.003806 | 0.033499107 | | 0.73 | 0.52 - 1.01 | 0.058011 | 0.216592159 |
| rs668626 | 1.67 | 1.13 - 2.46 | 0.009356 | 0.003881965 | | 1.51 | 1.05 - 2.18 | 0.026881 | 0.05749943 |
| rs798766 | 0.62 | 0.45 - 0.86 | 0.003969 | 0.048794396 | | 0.69 | 0.5 - 0.95 | 0.021974 | 0.219911842 |
| rs11621926 | 0.62 | 0.45 - 0.86 | 0.003984 | 0.015581798 | | 0.63 | 0.45 - 0.86 | 0.004114 | 0.063065244 |
| rs11646387 | 0.65 | 0.46 - 0.93 | 0.019482 | 0.0039887 | | 0.70 | 0.5 - 1 | 0.048695 | 0.006418626 |
| rs10507441 | 1.80 | 1.21 - 2.7 | 0.004057 | 0.048386383 | | 1.35 | 0.89 - 2.03 | 0.155188 | 0.289179598 |
| rs1046844 | 0.69 | 0.5 - 0.96 | 0.028949 | 0.004338851 | | 0.73 | 0.53 - 1 | 0.052823 | 0.010895287 |
| rs8139013 | 0.45 | 0.26 - 0.78 | 0.004375 | 0.024886329 | | 0.49 | 0.28 - 0.85 | 0.010799 | 0.022569447 |
| rs343169 | 0.27 | 0.11 - 0.66 | 0.004542 | 0.011553832 | | 0.35 | 0.15 - 0.81 | 0.014768 | 0.009212701 |
| rs1417609 | 1.86 | 1.21 - 2.86 | 0.004619 | 0.033758609 | | 1.51 | 0.96 - 2.4 | 0.07778 | 0.244973834 |
| rs17746918 | 0.62 | 0.42 - 0.92 | 0.01714 | 0.004652845 | | 0.65 | 0.45 - 0.96 | 0.0293 | 0.007904604 |
| rs5753454 | 0.61 | 0.44 - 0.86 | 0.004726 | 0.044469158 | | 0.70 | 0.51 - 0.98 | 0.036816 | 0.629895829 |
| rs17008403 | 1.69 | 1.17 - 2.44 | 0.004768 | 0.008010341 | | 1.55 | 1.08 - 2.24 | 0.01743 | 0.019558847 |
| rs769950 | 0.62 | 0.44 - 0.86 | 0.004817 | 0.111056964 | | 0.75 | 0.54 - 1.03 | 0.074473 | 0.478341966 |
