# Supplementary material for: TP53-based interaction analysis identifies cis-eQTL variants for TP53BP2, FBXO28, and FAM53A that associate with survival and treatment outcome in breast cancer
Source: Oncotarget. 2017 Feb 5;8(11):18381–98. doi: 10.18632/oncotarget.15110 (PMC5392336; doi:10.18632/oncotarget.15110)
Supplement: Supplementary file 2 [file oncotarget-08-18381-s002.docx]

**Supplementary Table 2.** Complete list of SNP:TP53 and SNP:Anthracycline interaction test p-values from the BCAC interaction analyses of the 136 SNPs selected based on the HEBCS-GWS pilot study. Both unadjusted and corresponding Benjamini-Hochberg adjusted p-values are shown.

|  |  | **Raw p-values (interaction test, age-adjusted)** | | | | | **Adjusted p-values (Benjamini-Hochberg)** | | | | |
| --- | --- | --- | --- | --- | --- | --- | --- | --- | --- | --- | --- |
|  |  | **P53** | **P53** | **P53** | **Anthrac.** | **Anthrac.** | **P53** | **P53** | **P53** | **Anthrac.** | **Anthrac.** |
| **SNP** | **Pos (hg18)** | **All cases** | **ER+** | **ER-** | **Additive** | **Recessive** | **All cases** | **ER+** | **ER-** | **Additive** | **Recessive** |
| rs10916264 | chr1: 222327447 | 0.01920166 | 3.44E-05 | 0.187722 | 0.025342 | 0.1098 | 0.217619 | 0.001135 | 0.94436 | 0.677354 | 0.711088 |
| rs6692043 | chr1: 222335061 | 0.01920166 | 3.44E-05 | 0.187722 | 0.025721 | 0.040992 | 0.217619 | 0.001135 | 0.94436 | 0.677354 | 0.711088 |
| rs6683927 | chr1: 222335725 | 0.01920166 | 3.44E-05 | 0.187722 | 0.032392 | 0.044848 | 0.217619 | 0.001135 | 0.94436 | 0.677354 | 0.711088 |
| rs6604887 | chr1: 222335577 | 0.01211554 | 4.14E-05 | 0.209445 | 0.021778 | 0.051215 | 0.217619 | 0.001135 | 0.94436 | 0.677354 | 0.711088 |
| rs10753443 | chr1: 222330791 | 0.01230473 | 4.17E-05 | 0.209445 | 0.029331 | 0.078101 | 0.217619 | 0.001135 | 0.94436 | 0.677354 | 0.711088 |
| rs798755 | chr4: 1690622 | 0.89868934 | 0.338807 | 0.16089 | 0.006455 | 9.57E-05 | 0.947455 | 0.70889 | 0.94436 | 0.677354 | 0.013016 |
| rs798741 | chr4: 1682211 | 0.85040899 | 0.428334 | 0.274 | 0.044635 | 0.004414 | 0.917902 | 0.810178 | 0.94436 | 0.677354 | 0.200094 |
| rs12875736 | chr13: 26111768 | 0.00595537 | 0.02027 | 0.281572 | 0.77025 | 0.481438 | 0.217619 | 0.339621 | 0.94436 | 0.87295 | 0.839431 |
| rs3096337 | chr3: 197017729 | 0.00702512 | 0.056234 | 0.008737 | 0.98669 | 0.918445 | 0.217619 | 0.339621 | 0.654588 | 0.993374 | 0.952053 |
| rs1018123 | chr13: 26112914 | 0.00867715 | 0.043032 | 0.246255 | 0.431921 | 0.842578 | 0.217619 | 0.339621 | 0.94436 | 0.772781 | 0.928997 |
| rs11805965 | chr1: 119789945 | 0.02250688 | 0.65966 | 0.009626 | 0.534613 | 0.308992 | 0.235457 | 0.887183 | 0.654588 | 0.772781 | 0.839431 |
| rs2031541 | chr13: 42119530 | 0.01369795 | 0.082477 | 0.165917 | 0.240213 | 0.203626 | 0.217619 | 0.339621 | 0.94436 | 0.772781 | 0.839431 |
| rs6907188 | chr6: 150387730 | 0.01835277 | 0.030059 | 0.268841 | 0.456077 | 0.433888 | 0.217619 | 0.339621 | 0.94436 | 0.772781 | 0.839431 |
| rs1275993 | chr2: 26661922 | 0.01662019 | 0.027435 | 0.371022 | 0.247938 | 0.748144 | 0.217619 | 0.339621 | 0.947309 | 0.772781 | 0.910024 |
| rs1148962 | chr2: 26671510 | 0.01813886 | 0.058617 | 0.299324 | 0.343131 | 0.626576 | 0.217619 | 0.339621 | 0.94436 | 0.772781 | 0.886344 |
| rs4919741 | chr12: 51559187 | 0.11037046 | 0.796306 | 0.022751 | 0.450342 | 0.424355 | 0.698327 | 0.907767 | 0.810175 | 0.772781 | 0.839431 |
| rs7739790 | chr6: 37281771 | 0.05242871 | 0.024831 | 0.682465 | 0.725076 | 0.632172 | 0.445644 | 0.339621 | 0.947309 | 0.83568 | 0.886344 |
| rs1417609 | chr1: 119779329 | 0.02918169 | 0.402641 | 0.024006 | 0.643203 | 0.631115 | 0.283479 | 0.805042 | 0.810175 | 0.801924 | 0.886344 |
| rs12665798 | chr6: 70492805 | 0.21516006 | 0.929339 | 0.282476 | 0.172205 | 0.097465 | 0.698327 | 0.96481 | 0.94436 | 0.772781 | 0.711088 |
| rs10764990 | chr10: 129042598 | 0.30690463 | 0.883859 | 0.032848 | 0.591488 | 0.927881 | 0.698327 | 0.946495 | 0.810175 | 0.783709 | 0.952053 |
| rs7551796 | chr1: 3308193 | 0.11483755 | 0.041819 | 0.696699 | 0.83405 | 0.720868 | 0.698327 | 0.339621 | 0.947309 | 0.922201 | 0.905828 |
| rs745052 | chr3: 67669015 | 0.50280084 | 0.793494 | 0.035743 | 0.559474 | 0.555632 | 0.777056 | 0.907767 | 0.810175 | 0.772781 | 0.872956 |
| rs4769504 | chr13: 26028516 | 0.04835961 | 0.076057 | 0.27801 | 0.38835 | 0.105642 | 0.43846 | 0.339621 | 0.94436 | 0.772781 | 0.711088 |
| rs1830115 | chr15: 21200233 | 0.27558778 | 0.775773 | 0.047407 | 0.46961 | 0.004008 | 0.698327 | 0.907767 | 0.85529 | 0.772781 | 0.200094 |
| rs10210979 | chr2: 238675991 | 0.8753878 | 0.048449 | 0.342997 | 0.416281 | 0.943382 | 0.9301 | 0.339621 | 0.947309 | 0.772781 | 0.952053 |
| rs12618367 | chr2: 45150469 | 0.32795087 | 0.750234 | 0.050311 | 0.608861 | 0.048398 | 0.698327 | 0.896471 | 0.85529 | 0.78862 | 0.711088 |
| rs11672071 | chr19: 13535239 | 0.18998773 | 0.055739 | 0.866047 | 0.85593 | 0.553152 | 0.698327 | 0.339621 | 0.947309 | 0.931252 | 0.872956 |
| rs2955092 | chr3: 129455218 | 0.33344447 | 0.058623 | 0.836196 | 0.348191 | 0.393396 | 0.698327 | 0.339621 | 0.947309 | 0.772781 | 0.839431 |
| rs11709066 | chr3: 129357602 | 0.32454798 | 0.060827 | 0.906802 | 0.35077 | 0.254679 | 0.698327 | 0.339621 | 0.947309 | 0.772781 | 0.839431 |
| rs10783548 | chr12: 51562179 | 0.13085393 | 0.536522 | 0.076084 | 0.779747 | 0.246102 | 0.698327 | 0.838702 | 0.94436 | 0.87641 | 0.839431 |
| rs12484656 | chr22: 41928325 | 0.39840174 | 0.875648 | 0.244349 | 0.450176 | 0.078248 | 0.698327 | 0.945143 | 0.94436 | 0.772781 | 0.711088 |
| rs2955129 | chr3: 129411897 | 0.35454537 | 0.065145 | 0.867964 | 0.324854 | 0.415119 | 0.698327 | 0.339621 | 0.947309 | 0.772781 | 0.839431 |
| rs2687730 | chr3: 129398147 | 0.35454537 | 0.065145 | 0.867964 | 0.325018 | 0.415119 | 0.698327 | 0.339621 | 0.947309 | 0.772781 | 0.839431 |
| rs11715661 | chr3: 129303536 | 0.32945209 | 0.070883 | 0.88115 | 0.349092 | 0.275367 | 0.698327 | 0.339621 | 0.947309 | 0.772781 | 0.839431 |
| rs11714052 | chr3: 129297147 | 0.32945209 | 0.070883 | 0.88115 | 0.361333 | 0.275549 | 0.698327 | 0.339621 | 0.947309 | 0.772781 | 0.839431 |
| rs11707462 | chr3: 129321320 | 0.32945209 | 0.070883 | 0.88115 | 0.383172 | 0.523987 | 0.698327 | 0.339621 | 0.947309 | 0.772781 | 0.872956 |
| rs10934850 | chr3: 129369647 | 0.40185421 | 0.074169 | 0.824347 | 0.32583 | 0.414894 | 0.698327 | 0.339621 | 0.947309 | 0.772781 | 0.839431 |
| rs11721213 | chr3: 129550131 | 0.31252855 | 0.074379 | 0.945569 | 0.564346 | 0.543441 | 0.698327 | 0.339621 | 0.959682 | 0.772781 | 0.872956 |
| rs10876347 | chr12: 51475307 | 0.08939154 | 0.153921 | 0.462147 | 0.095244 | 0.095572 | 0.698327 | 0.402563 | 0.947309 | 0.772781 | 0.711088 |
| rs1928564 | chr3: 67734693 | 0.52278605 | 0.815816 | 0.17983 | 0.119618 | 0.073337 | 0.794196 | 0.916951 | 0.94436 | 0.772781 | 0.711088 |
| rs7460206 | chr8: 80520868 | 0.64585672 | 0.238258 | 0.097677 | 0.62195 | 0.847026 | 0.850794 | 0.568475 | 0.94436 | 0.791018 | 0.928997 |
| rs7605254 | chr2: 239027677 | 0.45144301 | 0.081592 | 0.940516 | 0.985986 | 0.391041 | 0.739714 | 0.339621 | 0.959682 | 0.993374 | 0.839431 |
| rs2237172 | chr6: 16709566 | 0.16272767 | 0.660495 | 0.096412 | 0.297981 | 0.080418 | 0.698327 | 0.887183 | 0.94436 | 0.772781 | 0.711088 |
| rs2811400 | chr3: 129470557 | 0.40887315 | 0.093234 | 0.801823 | 0.325468 | 0.446375 | 0.698327 | 0.339621 | 0.947309 | 0.772781 | 0.839431 |
| rs2811373 | chr3: 129480119 | 0.38207257 | 0.09474 | 0.812242 | 0.268954 | 0.201069 | 0.698327 | 0.339621 | 0.947309 | 0.772781 | 0.839431 |
| rs7374952 | chr3: 129484057 | 0.38670189 | 0.096335 | 0.812242 | 0.349029 | 0.400647 | 0.698327 | 0.339621 | 0.947309 | 0.772781 | 0.839431 |
| rs7374227 | chr3: 129484205 | 0.38670189 | 0.096335 | 0.812242 | 0.347326 | 0.400542 | 0.698327 | 0.339621 | 0.947309 | 0.772781 | 0.839431 |
| rs2955094 | chr3: 129459613 | 0.4056341 | 0.096335 | 0.83853 | 0.350233 | 0.400524 | 0.698327 | 0.339621 | 0.947309 | 0.772781 | 0.839431 |
| rs2811544 | chr3: 129466185 | 0.4056341 | 0.096335 | 0.83853 | 0.350233 | 0.400524 | 0.698327 | 0.339621 | 0.947309 | 0.772781 | 0.839431 |
| rs2811538 | chr3: 129469321 | 0.4056341 | 0.096335 | 0.83853 | 0.350233 | 0.400524 | 0.698327 | 0.339621 | 0.947309 | 0.772781 | 0.839431 |
| rs2811533 | chr3: 129474419 | 0.4056341 | 0.096335 | 0.83853 | 0.351578 | 0.480494 | 0.698327 | 0.339621 | 0.947309 | 0.772781 | 0.839431 |
| rs2811519 | chr3: 129495566 | 0.38670189 | 0.096335 | 0.812242 | 0.348396 | 0.400524 | 0.698327 | 0.339621 | 0.947309 | 0.772781 | 0.839431 |
| rs2811518 | chr3: 129496335 | 0.38670189 | 0.096335 | 0.812242 | 0.348396 | 0.47643 | 0.698327 | 0.339621 | 0.947309 | 0.772781 | 0.839431 |
| rs2811527 | chr3: 129477294 | 0.41031301 | 0.097631 | 0.83853 | 0.396433 | 0.400547 | 0.698327 | 0.339621 | 0.947309 | 0.772781 | 0.839431 |
| rs575381 | chr20: 53083555 | 0.62974825 | 0.495113 | 0.545891 | 0.068496 | 0.809666 | 0.850794 | 0.8313 | 0.947309 | 0.772781 | 0.928997 |
| rs11621926 | chr14: 95124015 | 0.16141268 | 0.100601 | 0.817449 | 0.493192 | 0.899386 | 0.698327 | 0.339621 | 0.947309 | 0.772781 | 0.950603 |
| rs1542287 | chr16: 8366510 | 0.5827789 | 0.842808 | 0.138926 | 0.309479 | 0.672101 | 0.825603 | 0.931886 | 0.94436 | 0.772781 | 0.891266 |
| rs2673515 | chr3: 21869752 | 0.48860078 | 0.826656 | 0.809436 | 0.081168 | 0.187836 | 0.76379 | 0.921518 | 0.947309 | 0.772781 | 0.839431 |
| rs6439124 | chr3: 129490156 | 0.4053424 | 0.103391 | 0.812242 | 0.359819 | 0.399737 | 0.698327 | 0.339621 | 0.947309 | 0.772781 | 0.839431 |
| rs2955096 | chr3: 129460556 | 0.41591517 | 0.103866 | 0.866103 | 0.450189 | 0.476563 | 0.698327 | 0.339621 | 0.947309 | 0.772781 | 0.839431 |
| rs10507441 | chr13: 36231087 | 0.30400004 | 0.552514 | 0.249694 | 0.136025 | 0.142807 | 0.698327 | 0.853885 | 0.94436 | 0.772781 | 0.839431 |
| rs9345095 | chr6: 91894885 | 0.36372824 | 0.64944 | 0.333304 | 0.241949 | 0.285755 | 0.698327 | 0.887183 | 0.94436 | 0.772781 | 0.839431 |
| rs7968748 | chr12: 51482318 | 0.1589458 | 0.159576 | 0.738633 | 0.078761 | 0.500738 | 0.698327 | 0.409478 | 0.947309 | 0.772781 | 0.86203 |
| rs9925768 | chr16: 83735148 | 0.67789563 | 0.442732 | 0.872322 | 0.042368 | 0.07572 | 0.850794 | 0.812251 | 0.947309 | 0.677354 | 0.711088 |
| rs3947930 | chr6: 70565041 | 0.22195429 | 0.942341 | 0.279949 | 0.15932 | 0.678668 | 0.698327 | 0.970897 | 0.94436 | 0.772781 | 0.891266 |
| rs2811416 | chr3: 129474628 | 0.47307869 | 0.113157 | 0.798022 | 0.360962 | 0.53843 | 0.748124 | 0.339621 | 0.947309 | 0.772781 | 0.872956 |
| rs12533185 | chr7: 8730890 | 0.19509409 | 0.703808 | 0.406723 | 0.077521 | 0.415459 | 0.698327 | 0.887183 | 0.947309 | 0.772781 | 0.839431 |
| rs13227862 | chr7: 116604668 | 0.52574628 | 0.120783 | 0.223446 | 0.686153 | 0.712071 | 0.794196 | 0.339621 | 0.94436 | 0.808348 | 0.905828 |
| rs17360838 | chr4: 153108496 | 0.82614328 | 0.508344 | 0.116253 | 0.49544 | 0.41553 | 0.917902 | 0.83295 | 0.94436 | 0.772781 | 0.839431 |
| rs8139013 | chr22: 41610003 | 0.12664056 | 0.289187 | 0.465058 | 0.226297 | 0.814778 | 0.698327 | 0.644745 | 0.947309 | 0.772781 | 0.928997 |
| rs16844002 | chr3: 129536177 | 0.40986507 | 0.121274 | 0.854698 | 0.482524 | 0.447712 | 0.698327 | 0.339621 | 0.947309 | 0.772781 | 0.839431 |
| rs6798749 | chr3: 129539587 | 0.39303878 | 0.123303 | 0.919447 | 0.503188 | 0.44501 | 0.698327 | 0.339621 | 0.947309 | 0.772781 | 0.839431 |
| rs11710704 | chr3: 129529926 | 0.39728688 | 0.123303 | 0.902756 | 0.4947 | 0.445044 | 0.698327 | 0.339621 | 0.947309 | 0.772781 | 0.839431 |
| rs2037965 | chr3: 129507734 | 0.40189181 | 0.124861 | 0.902756 | 0.524212 | 0.670976 | 0.698327 | 0.339621 | 0.947309 | 0.772781 | 0.891266 |
| rs16843876 | chr3: 129515230 | 0.40189181 | 0.124861 | 0.902756 | 0.527378 | 0.674682 | 0.698327 | 0.339621 | 0.947309 | 0.772781 | 0.891266 |
| rs11706826 | chr3: 129515681 | 0.40189181 | 0.124861 | 0.902756 | 0.512891 | 0.418062 | 0.698327 | 0.339621 | 0.947309 | 0.772781 | 0.839431 |
| rs6595079 | chr5: 117343624 | 0.22497573 | 0.124618 | 0.452343 | 0.526967 | 0.285801 | 0.698327 | 0.339621 | 0.947309 | 0.772781 | 0.839431 |
| rs2811388 | chr3: 129501137 | 0.41436552 | 0.131275 | 0.902756 | 0.451154 | 0.383697 | 0.698327 | 0.350066 | 0.947309 | 0.772781 | 0.839431 |
| rs2178373 | chr6: 91858606 | 0.31729805 | 0.611266 | 0.31692 | 0.287258 | 0.343187 | 0.698327 | 0.887183 | 0.94436 | 0.772781 | 0.839431 |
| rs760645 | chr22: 37362216 | 0.2159922 | 0.662045 | 0.140875 | 0.440213 | 0.74284 | 0.698327 | 0.887183 | 0.94436 | 0.772781 | 0.910024 |
| rs12078739 | chr1: 235621161 | 0.15010153 | 0.256032 | 0.727031 | 0.50144 | 0.551509 | 0.698327 | 0.590175 | 0.947309 | 0.772781 | 0.872956 |
| rs1885277 | chr1: 67296372 | 0.68188647 | 0.386008 | 0.164901 | 0.678294 | 0.422296 | 0.850794 | 0.783538 | 0.94436 | 0.808348 | 0.839431 |
| rs11646387 | chr16: 10310261 | 0.17117642 | 0.321765 | 0.353392 | 0.243122 | 0.592035 | 0.698327 | 0.694604 | 0.947309 | 0.772781 | 0.886344 |
| rs2570069 | chr4: 58851608 | 0.77655832 | 0.46585 | 0.915982 | 0.184562 | 0.11509 | 0.887588 | 0.812251 | 0.947309 | 0.772781 | 0.711465 |
| rs4856867 | chr3: 67632128 | 0.60842689 | 0.678626 | 0.15306 | 0.66175 | 0.516445 | 0.835819 | 0.887183 | 0.94436 | 0.808348 | 0.872956 |
| rs2076546 | chr20: 45701900 | 0.25875459 | 0.230222 | 0.571359 | 0.213681 | 0.23609 | 0.698327 | 0.55911 | 0.947309 | 0.772781 | 0.839431 |
| rs2634734 | chr3: 67727937 | 0.53141087 | 0.796703 | 0.164608 | 0.138018 | 0.087261 | 0.794196 | 0.907767 | 0.94436 | 0.772781 | 0.711088 |
| rs2276881 | chr4: 3201459 | 0.15891887 | 0.421881 | 0.304006 | 0.882995 | 0.164922 | 0.698327 | 0.810178 | 0.94436 | 0.945569 | 0.839431 |
| rs4878743 | chr9: 38048424 | 0.92558373 | 0.178446 | 0.235069 | 0.993374 | 0.908665 | 0.966003 | 0.449419 | 0.94436 | 0.993374 | 0.950603 |
| rs904816 | chr16: 83737161 | 0.60157972 | 0.46332 | 0.739139 | 0.049805 | 0.105755 | 0.835819 | 0.812251 | 0.947309 | 0.677354 | 0.711088 |
| rs11864373 | chr16: 83736591 | 0.66565255 | 0.531929 | 0.743559 | 0.047971 | 0.078186 | 0.850794 | 0.838702 | 0.947309 | 0.677354 | 0.711088 |
| rs13099918 | chr3: 4320766 | 0.66457278 | 0.684583 | 0.202844 | 0.146132 | 0.76221 | 0.850794 | 0.887183 | 0.94436 | 0.772781 | 0.91735 |
| rs1866084 | chr8: 85202511 | 0.71317852 | 0.655724 | 0.289155 | 0.593544 | 0.324264 | 0.880239 | 0.887183 | 0.94436 | 0.783709 | 0.839431 |
| rs343169 | chr4: 122035537 | 0.20199336 | 0.701564 | 0.212061 | 0.92612 | 0.973934 | 0.698327 | 0.887183 | 0.94436 | 0.968864 | 0.973934 |
| rs10908367 | chr1: 37907193 | 0.28079956 | 0.221525 | 0.569146 | 0.648615 | 0.83448 | 0.698327 | 0.547772 | 0.947309 | 0.801924 | 0.928997 |
| rs12693085 | chr2: 176446786 | 0.65079662 | 0.536503 | 0.76972 | 0.34961 | 0.562132 | 0.850794 | 0.838702 | 0.947309 | 0.772781 | 0.872956 |
| rs12444778 | chr16: 80330728 | 0.77663992 | 0.709956 | 0.208537 | 0.525356 | 0.064115 | 0.887588 | 0.887183 | 0.94436 | 0.772781 | 0.711088 |
| rs750358 | chr15: 25701843 | 0.31544501 | 0.793711 | 0.97453 | 0.573904 | 0.624894 | 0.698327 | 0.907767 | 0.97453 | 0.772781 | 0.886344 |
| rs7080287 | chr10: 115094788 | 0.99543313 | 0.428918 | 0.68888 | 0.900189 | 0.86626 | 0.995433 | 0.810178 | 0.947309 | 0.949037 | 0.935011 |
| rs310834 | chr12: 75934979 | 0.84087358 | 0.861812 | 0.707563 | 0.971439 | 0.249269 | 0.917902 | 0.945143 | 0.947309 | 0.993351 | 0.839431 |
| rs7947323 | chr11: 85789111 | 0.95915835 | 0.723058 | 0.660666 | 0.108177 | 0.627392 | 0.987486 | 0.888331 | 0.947309 | 0.772781 | 0.886344 |
| rs5753454 | chr22: 29805307 | 0.97700127 | 0.253831 | 0.427736 | 0.622345 | 0.463513 | 0.987486 | 0.590175 | 0.947309 | 0.791018 | 0.839431 |
| rs11711870 | chr3: 197034763 | 0.26034015 | 0.961902 | 0.851845 | 0.412425 | 0.470927 | 0.698327 | 0.976259 | 0.947309 | 0.772781 | 0.839431 |
| rs9604787 | chr22: 16614589 | 0.74653168 | 0.711051 | 0.315261 | 0.29216 | 0.449652 | 0.887588 | 0.887183 | 0.94436 | 0.772781 | 0.839431 |
| rs7760914 | chr6: 151649195 | 0.74233744 | 0.263539 | 0.261262 | 0.484694 | 0.209672 | 0.887588 | 0.597355 | 0.94436 | 0.772781 | 0.839431 |
| rs13269443 | chr8: 85196126 | 0.81226988 | 0.725035 | 0.4111 | 0.530135 | 0.725994 | 0.917902 | 0.888331 | 0.947309 | 0.772781 | 0.905828 |
| rs7341568 | chr8: 85189484 | 0.85025871 | 0.691281 | 0.4111 | 0.521557 | 0.724243 | 0.917902 | 0.887183 | 0.947309 | 0.772781 | 0.905828 |
| rs350784 | chr2: 52787649 | 0.30317622 | 0.408441 | 0.404177 | 0.542369 | 0.44121 | 0.698327 | 0.805042 | 0.947309 | 0.772781 | 0.839431 |
| rs10481281 | chr8: 85211245 | 0.84164906 | 0.607693 | 0.324037 | 0.635389 | 0.945053 | 0.917902 | 0.887183 | 0.94436 | 0.80012 | 0.952053 |
| rs1133603 | chr3: 118133470 | 0.33327993 | 0.956756 | 0.574946 | 0.463081 | 0.443451 | 0.698327 | 0.976259 | 0.947309 | 0.772781 | 0.839431 |
| rs2279290 | chr3: 66513501 | 0.97489329 | 0.326879 | 0.658271 | 0.543971 | 0.616021 | 0.987486 | 0.694617 | 0.947309 | 0.772781 | 0.886344 |
| rs970694 | chr4: 92969417 | 0.71843062 | 0.701944 | 0.381704 | 0.819229 | 0.83975 | 0.880239 | 0.887183 | 0.947309 | 0.913239 | 0.928997 |
| rs11155550 | chr6: 148474003 | 0.29848442 | 0.320132 | 0.68314 | 0.873965 | 0.318203 | 0.698327 | 0.694604 | 0.947309 | 0.943328 | 0.839431 |
| rs6886725 | chr5: 124297257 | 0.46025742 | 0.615771 | 0.663272 | 0.665992 | 0.174285 | 0.745179 | 0.887183 | 0.947309 | 0.808348 | 0.839431 |
| rs12494912 | chr3: 66534597 | 0.29249977 | 0.928765 | 0.430511 | 0.360106 | 0.425834 | 0.698327 | 0.96481 | 0.947309 | 0.772781 | 0.839431 |
| rs1874786 | chr6: 92632039 | 0.83200992 | 0.703315 | 0.376689 | 0.444266 | 0.612334 | 0.917902 | 0.887183 | 0.947309 | 0.772781 | 0.886344 |
| rs10934370 | chr3: 118131380 | 0.37217733 | 0.971597 | 0.636058 | 0.536149 | 0.572728 | 0.698327 | 0.978794 | 0.947309 | 0.772781 | 0.87518 |
| rs7702447 | chr5: 31993307 | 0.86023716 | 0.3685 | 0.910131 | 0.468613 | 0.249081 | 0.921199 | 0.759334 | 0.947309 | 0.772781 | 0.839431 |
| rs12632280 | chr3: 87804944 | 0.5730799 | 0.74542 | 0.332941 | 0.491293 | 0.810659 | 0.820409 | 0.896471 | 0.94436 | 0.772781 | 0.928997 |
| rs606410 | chr13: 76958044 | 0.93048807 | 0.874174 | 0.602657 | 0.171371 | 0.077617 | 0.966003 | 0.945143 | 0.947309 | 0.772781 | 0.711088 |
| rs179734 | chr14: 30560806 | 0.33856867 | 0.488663 | 0.445617 | 0.463503 | 0.749432 | 0.698327 | 0.830727 | 0.947309 | 0.772781 | 0.910024 |
| rs4720305 | chr7: 4484636 | 0.60355961 | 0.486294 | 0.728449 | 0.554577 | 0.940121 | 0.835819 | 0.830727 | 0.947309 | 0.772781 | 0.952053 |
| rs2158232 | chr16: 17862049 | 0.55613433 | 0.448681 | 0.866503 | 0.425931 | 0.814748 | 0.813272 | 0.812251 | 0.947309 | 0.772781 | 0.928997 |
| rs7621006 | chr3: 32689621 | 0.44553089 | 0.655242 | 0.762456 | 0.898986 | 0.673553 | 0.738929 | 0.887183 | 0.947309 | 0.949037 | 0.891266 |
| rs7114163 | chr11: 11274524 | 0.76833173 | 0.800971 | 0.672027 | 0.67263 | 0.836887 | 0.887588 | 0.907767 | 0.947309 | 0.808348 | 0.928997 |
| rs170249 | chr14: 79139680 | 0.67327453 | 0.446181 | 0.665729 | 0.385214 | 0.427252 | 0.850794 | 0.812251 | 0.947309 | 0.772781 | 0.839431 |
| rs515028 | chr10: 115101082 | 0.57108098 | 0.453912 | 0.598732 | 0.943493 | 0.821561 | 0.820409 | 0.812251 | 0.947309 | 0.979504 | 0.928997 |
| rs11920441 | chr3: 51075060 | 0.46893801 | 0.927037 | 0.463399 | 0.441239 | 0.332499 | 0.748124 | 0.96481 | 0.947309 | 0.772781 | 0.839431 |
| rs644184 | chr3: 29088197 | 0.53841737 | 0.751454 | 0.969437 | 0.572256 | 0.875966 | 0.795921 | 0.896471 | 0.97453 | 0.772781 | 0.938042 |
| rs12613687 | chr2: 200707501 | 0.41013539 | 0.903563 | 0.586393 | 0.853784 | 0.643395 | 0.698327 | 0.960036 | 0.947309 | 0.931252 | 0.891266 |
| rs950590 | chr4: 186891371 | 0.74553404 | 0.505146 | 0.674945 | 0.571128 | 0.681556 | 0.887588 | 0.83295 | 0.947309 | 0.772781 | 0.891266 |
| rs17008403 | chr3: 71385139 | 0.77122996 | 0.527468 | 0.566003 | 0.601598 | 0.564854 | 0.887588 | 0.838702 | 0.947309 | 0.786705 | 0.872956 |
| rs11822684 | chr11: 109989463 | 0.98022463 | 0.705069 | 0.879957 | 0.702037 | 0.90731 | 0.987486 | 0.887183 | 0.947309 | 0.816043 | 0.950603 |
| rs2282328 | chr6: 132801618 | 0.63833746 | 0.647511 | 0.557612 | 0.952402 | 0.705202 | 0.850794 | 0.887183 | 0.947309 | 0.981263 | 0.905828 |
| rs1546010 | chr18: 62496449 | 0.65521697 | 0.698525 | 0.784626 | 0.689473 | 0.854206 | 0.850794 | 0.887183 | 0.947309 | 0.808348 | 0.929376 |
| rs603682 | chr3: 29109113 | 0.77479012 | 0.995332 | 0.762806 | 0.740489 | 0.790246 | 0.887588 | 0.995332 | 0.947309 | 0.846273 | 0.928997 |
