# Supplementary material for: TP53-based interaction analysis identifies cis-eQTL variants for TP53BP2, FBXO28, and FAM53A that associate with survival and treatment outcome in breast cancer
Source: Oncotarget. 2017 Feb 5;8(11):18381–98. doi: 10.18632/oncotarget.15110 (PMC5392336; doi:10.18632/oncotarget.15110)
Supplement: Supplementary file 3 [file oncotarget-08-18381-s003.docx]

**Supplementary Table 3.** List of all BCAC SNPs and their linkage disequilibrium with the corresponding SNPs in the HEBCS-GWS pilot. Note that duplicate lines occur for many SNPs due to multiple SNPs tagging the SNP in the other data set.

| **HEBCS SNP** | **R^2^** | **D'** | **BCAC SNP** |
| --- | --- | --- | --- |
| rs7986966 | 1 | 1 | rs1018123 |
| rs1018123 | 1 | 1 | rs1018123 |
| rs10210979 | 1 | 1 | rs10210979 |
| rs10481281 | 1 | 1 | rs10481281 |
| rs10507441 | 1 | 1 | rs10507441 |
| rs1388970 | 1 | 1 | rs10753443 |
| rs6604887 | 1 | 1 | rs10753443 |
| rs10764990 | 1 | 1 | rs10764990 |
| rs4919741 | 0.885 | 1 | rs10783548 |
| rs10876347 | 1 | 1 | rs10876347 |
| rs10908367 | 1 | 1 | rs10908367 |
| rs1388970 | 0.934 | 0.966 | rs10916264 |
| rs6604887 | 0.934 | 0.966 | rs10916264 |
| rs1133603 | 0.947 | 1 | rs10934370 |
| rs1523262 | 0.895 | 1 | rs10934370 |
| rs10934370 | 1 | 1 | rs10934370 |
| rs6798749 | 1 | 1 | rs10934850 |
| rs2811388 | 0.908 | 1 | rs10934850 |
| rs2999081 | 0.908 | 1 | rs10934850 |
| rs2811518 | 1 | 1 | rs10934850 |
| rs11720239 | 1 | 1 | rs10934850 |
| rs11155550 | 1 | 1 | rs11155550 |
| rs1133603 | 1 | 1 | rs1133603 |
| rs1523262 | 0.945 | 1 | rs1133603 |
| rs10934370 | 0.947 | 1 | rs1133603 |
| rs1275993 | 1 | 1 | rs1148962 |
| rs11621926 | 1 | 1 | rs11621926 |
| rs11646387 | 1 | 1 | rs11646387 |
| rs11672071 | 1 | 1 | rs11672071 |
| rs6798749 | 1 | 1 | rs11706826 |
| rs2811388 | 0.908 | 1 | rs11706826 |
| rs2999081 | 0.908 | 1 | rs11706826 |
| rs2811518 | 1 | 1 | rs11706826 |
| rs11720239 | 1 | 1 | rs11706826 |
| rs6798749 | 1 | 1 | rs11707462 |
| rs2811388 | 0.908 | 1 | rs11707462 |
| rs2999081 | 0.908 | 1 | rs11707462 |
| rs2811518 | 1 | 1 | rs11707462 |
| rs11720239 | 1 | 1 | rs11707462 |
| rs6798749 | 1 | 1 | rs11709066 |
| rs2811388 | 0.908 | 1 | rs11709066 |
| rs2999081 | 0.908 | 1 | rs11709066 |
| rs2811518 | 1 | 1 | rs11709066 |
| rs11720239 | 1 | 1 | rs11709066 |
| rs6798749 | 1 | 1 | rs11710704 |
| rs2811388 | 0.908 | 1 | rs11710704 |
| rs2999081 | 0.908 | 1 | rs11710704 |
| rs2811518 | 1 | 1 | rs11710704 |
| rs11720239 | 1 | 1 | rs11710704 |
| rs11711870 | 1 | 1 | rs11711870 |
| rs6798749 | 1 | 1 | rs11714052 |
| rs2811388 | 0.908 | 1 | rs11714052 |
| rs2999081 | 0.908 | 1 | rs11714052 |
| rs2811518 | 1 | 1 | rs11714052 |
| rs11720239 | 1 | 1 | rs11714052 |
| rs6798749 | 0.901 | 1 | rs11715661 |
| rs2811388 | 0.818 | 1 | rs11715661 |
| rs2999081 | 0.818 | 1 | rs11715661 |
| rs2811518 | 0.901 | 1 | rs11715661 |
| rs11720239 | 0.901 | 1 | rs11715661 |
| rs6798749 | 1 | 1 | rs11721213 |
| rs2811388 | 0.908 | 1 | rs11721213 |
| rs2999081 | 0.908 | 1 | rs11721213 |
| rs2811518 | 1 | 1 | rs11721213 |
| rs11720239 | 1 | 1 | rs11721213 |
| rs1417609 | 0.841 | 1 | rs11805965 |
| rs11822684 | 1 | 1 | rs11822684 |
| rs11864373 | 1 | 1 | rs11864373 |
| rs9925768 | 1 | 1 | rs11864373 |
| rs11920441 | 1 | 1 | rs11920441 |
| rs11928389 | 0.966 | 1 | rs11920441 |
| rs12078739 | 1 | 1 | rs12078739 |
| rs12444778 | 1 | 1 | rs12444778 |
| rs12484656 | 1 | 1 | rs12484656 |
| rs12494912 | 1 | 1 | rs12494912 |
| rs1046844 | 1 | 1 | rs12494912 |
| rs12533185 | 1 | 1 | rs12533185 |
| rs12613687 | 1 | 1 | rs12613687 |
| rs769950 | 0.951 | 1 | rs12613687 |
| rs12618367 | 1 | 1 | rs12618367 |
| rs4643731 | 0.961 | 1 | rs12632280 |
| rs1509778 | 0.961 | 1 | rs12632280 |
| rs2445958 | 0.967 | 1 | rs12665798 |
| rs12693085 | 1 | 1 | rs12693085 |
| rs1275993 | 1 | 1 | rs1275993 |
| rs7986966 | 0.839 | 1 | rs12875736 |
| rs1018123 | 0.839 | 1 | rs12875736 |
| rs13099918 | 1 | 1 | rs13099918 |
| rs13227862 | 1 | 1 | rs13227862 |
| rs10481281 | 1 | 1 | rs13269443 |
| rs1417609 | 1 | 1 | rs1417609 |
| rs1542287 | 1 | 1 | rs1542287 |
| rs9941427 | 1 | 1 | rs1546010 |
| rs1546010 | 1 | 1 | rs1546010 |
| rs6798749 | 0.901 | 1 | rs16843876 |
| rs2811388 | 0.818 | 1 | rs16843876 |
| rs2999081 | 0.818 | 1 | rs16843876 |
| rs2811518 | 0.901 | 1 | rs16843876 |
| rs11720239 | 0.901 | 1 | rs16843876 |
| rs6798749 | 1 | 1 | rs16844002 |
| rs2811388 | 0.908 | 1 | rs16844002 |
| rs2999081 | 0.908 | 1 | rs16844002 |
| rs2811518 | 1 | 1 | rs16844002 |
| rs11720239 | 1 | 1 | rs16844002 |
| rs17008403 | 1 | 1 | rs17008403 |
| rs221417 | 1 | 1 | rs170249 |
| rs2543578 | 1 | 1 | rs170249 |
| rs17360838 | 1 | 1 | rs17360838 |
| rs7155894 | 1 | 1 | rs179734 |
| rs179734 | 1 | 1 | rs179734 |
| rs1830115 | 1 | 1 | rs1830115 |
| rs10481281 | 1 | 1 | rs1866084 |
| rs1874786 | 1 | 1 | rs1874786 |
| rs1885277 | 1 | 1 | rs1885277 |
| rs2634734 | 1 | 1 | rs1928564 |
| rs4941434 | 0.933 | 1 | rs2031541 |
| rs6798749 | 1 | 1 | rs2037965 |
| rs2811388 | 0.908 | 1 | rs2037965 |
| rs2999081 | 0.908 | 1 | rs2037965 |
| rs2811518 | 1 | 1 | rs2037965 |
| rs11720239 | 1 | 1 | rs2037965 |
| rs6018611 | 0.915 | 1 | rs2076546 |
| rs2868802 | 1 | 1 | rs2076546 |
| rs6018564 | 1 | 1 | rs2076546 |
| rs2284322 | 1 | 1 | rs2076546 |
| rs6018492 | 1 | 1 | rs2076546 |
| rs4344726 | 0.902 | 1 | rs2158232 |
| rs4377765 | 1 | 1 | rs2178373 |
| rs2237172 | 1 | 1 | rs2237172 |
| rs2276881 | 1 | 1 | rs2276881 |
| rs12494912 | 0.911 | 1 | rs2279290 |
| rs1046844 | 0.911 | 1 | rs2279290 |
| rs9385608 | 1 | 1 | rs2282328 |
| rs7746504 | 1 | 1 | rs2282328 |
| rs2570069 | 1 | 1 | rs2570069 |
| rs2634734 | 1 | 1 | rs2634734 |
| rs2673521 | 0.913 | 1 | rs2673515 |
| rs6798749 | 1 | 1 | rs2687730 |
| rs2811388 | 0.908 | 1 | rs2687730 |
| rs2999081 | 0.908 | 1 | rs2687730 |
| rs2811518 | 1 | 1 | rs2687730 |
| rs11720239 | 1 | 1 | rs2687730 |
| rs6798749 | 0.908 | 1 | rs2811373 |
| rs2811388 | 0.822 | 0.907 | rs2811373 |
| rs2999081 | 1 | 1 | rs2811373 |
| rs2811518 | 0.908 | 1 | rs2811373 |
| rs11720239 | 0.908 | 1 | rs2811373 |
| rs6798749 | 0.908 | 1 | rs2811388 |
| rs2811388 | 1 | 1 | rs2811388 |
| rs2999081 | 0.822 | 0.907 | rs2811388 |
| rs2811518 | 0.908 | 1 | rs2811388 |
| rs11720239 | 0.908 | 1 | rs2811388 |
| rs6798749 | 0.901 | 1 | rs2811400 |
| rs2811388 | 0.818 | 1 | rs2811400 |
| rs2999081 | 0.818 | 1 | rs2811400 |
| rs2811518 | 0.901 | 1 | rs2811400 |
| rs11720239 | 0.901 | 1 | rs2811400 |
| rs6798749 | 1 | 1 | rs2811416 |
| rs2811388 | 0.908 | 1 | rs2811416 |
| rs2999081 | 0.908 | 1 | rs2811416 |
| rs2811518 | 1 | 1 | rs2811416 |
| rs11720239 | 1 | 1 | rs2811416 |
| rs6798749 | 1 | 1 | rs2811518 |
| rs2811388 | 0.908 | 1 | rs2811518 |
| rs2999081 | 0.908 | 1 | rs2811518 |
| rs2811518 | 1 | 1 | rs2811518 |
| rs11720239 | 1 | 1 | rs2811518 |
| rs6798749 | 1 | 1 | rs2811519 |
| rs2811388 | 0.908 | 1 | rs2811519 |
| rs2999081 | 0.908 | 1 | rs2811519 |
| rs2811518 | 1 | 1 | rs2811519 |
| rs11720239 | 1 | 1 | rs2811519 |
| rs6798749 | 1 | 1 | rs2811527 |
| rs2811388 | 0.908 | 1 | rs2811527 |
| rs2999081 | 0.908 | 1 | rs2811527 |
| rs2811518 | 1 | 1 | rs2811527 |
| rs11720239 | 1 | 1 | rs2811527 |
| rs6798749 | 1 | 1 | rs2811533 |
| rs2811388 | 0.908 | 1 | rs2811533 |
| rs2999081 | 0.908 | 1 | rs2811533 |
| rs2811518 | 1 | 1 | rs2811533 |
| rs11720239 | 1 | 1 | rs2811533 |
| rs6798749 | 1 | 1 | rs2811538 |
| rs2811388 | 0.908 | 1 | rs2811538 |
| rs2999081 | 0.908 | 1 | rs2811538 |
| rs2811518 | 1 | 1 | rs2811538 |
| rs11720239 | 1 | 1 | rs2811538 |
| rs6798749 | 1 | 1 | rs2811544 |
| rs2811388 | 0.908 | 1 | rs2811544 |
| rs2999081 | 0.908 | 1 | rs2811544 |
| rs2811518 | 1 | 1 | rs2811544 |
| rs11720239 | 1 | 1 | rs2811544 |
| rs6798749 | 0.908 | 1 | rs2955092 |
| rs2811388 | 0.822 | 0.907 | rs2955092 |
| rs2999081 | 0.822 | 0.907 | rs2955092 |
| rs2811518 | 0.908 | 1 | rs2955092 |
| rs11720239 | 0.908 | 1 | rs2955092 |
| rs6798749 | 0.908 | 1 | rs2955094 |
| rs2811388 | 0.822 | 0.907 | rs2955094 |
| rs2999081 | 0.822 | 0.907 | rs2955094 |
| rs2811518 | 0.908 | 1 | rs2955094 |
| rs11720239 | 0.908 | 1 | rs2955094 |
| rs6798749 | 1 | 1 | rs2955096 |
| rs2811388 | 0.908 | 1 | rs2955096 |
| rs2999081 | 0.908 | 1 | rs2955096 |
| rs2811518 | 1 | 1 | rs2955096 |
| rs11720239 | 1 | 1 | rs2955096 |
| rs6798749 | 1 | 1 | rs2955129 |
| rs2811388 | 0.908 | 1 | rs2955129 |
| rs2999081 | 0.908 | 1 | rs2955129 |
| rs2811518 | 1 | 1 | rs2955129 |
| rs11720239 | 1 | 1 | rs2955129 |
| rs3096337 | 1 | 1 | rs3096337 |
| rs310834 | 1 | 1 | rs310834 |
| rs343169 | 1 | 1 | rs343169 |
| rs350784 | 1 | 1 | rs350784 |
| rs2445958 | 0.967 | 1 | rs3947930 |
| rs4720305 | 1 | 1 | rs4720305 |
| rs498498 | 0.911 | 1 | rs4769504 |
| rs653920 | 0.911 | 1 | rs4769504 |
| rs9553874 | 0.911 | 1 | rs4769504 |
| rs4769504 | 1 | 1 | rs4769504 |
| rs9553864 | 1 | 1 | rs4769504 |
| rs7628838 | 0.961 | 1 | rs4856867 |
| rs6792584 | 0.925 | 1 | rs4856867 |
| rs4856867 | 1 | 1 | rs4856867 |
| rs776021 | 0.961 | 1 | rs4878743 |
| rs4919741 | 1 | 1 | rs4919741 |
| rs515028 | 1 | 1 | rs515028 |
| rs5753454 | 1 | 1 | rs5753454 |
| rs575381 | 1 | 1 | rs575381 |
| rs603682 | 1 | 1 | rs603682 |
| rs668626 | 0.959 | 1 | rs603682 |
| rs606410 | 1 | 1 | rs606410 |
| rs6798749 | 1 | 1 | rs6439124 |
| rs2811388 | 0.908 | 1 | rs6439124 |
| rs2999081 | 0.908 | 1 | rs6439124 |
| rs2811518 | 1 | 1 | rs6439124 |
| rs11720239 | 1 | 1 | rs6439124 |
| rs603682 | 0.836 | 0.954 | rs644184 |
| rs668626 | 0.801 | 0.953 | rs644184 |
| rs6595079 | 1 | 1 | rs6595079 |
| rs1388970 | 1 | 1 | rs6604887 |
| rs6604887 | 1 | 1 | rs6604887 |
| rs1388970 | 0.934 | 0.966 | rs6683927 |
| rs6604887 | 0.934 | 0.966 | rs6683927 |
| rs1388970 | 0.934 | 0.966 | rs6692043 |
| rs6604887 | 0.934 | 0.966 | rs6692043 |
| rs6798749 | 1 | 1 | rs6798749 |
| rs2811388 | 0.908 | 1 | rs6798749 |
| rs2999081 | 0.908 | 1 | rs6798749 |
| rs2811518 | 1 | 1 | rs6798749 |
| rs11720239 | 1 | 1 | rs6798749 |
| rs6886725 | 1 | 1 | rs6886725 |
| rs6907188 | 1 | 1 | rs6907188 |
| rs7080287 | 1 | 1 | rs7080287 |
| rs7114163 | 1 | 1 | rs7114163 |
| rs10481281 | 1 | 1 | rs7341568 |
| rs6798749 | 1 | 1 | rs7374227 |
| rs2811388 | 0.908 | 1 | rs7374227 |
| rs2999081 | 0.908 | 1 | rs7374227 |
| rs2811518 | 1 | 1 | rs7374227 |
| rs11720239 | 1 | 1 | rs7374227 |
| rs6798749 | 1 | 1 | rs7374952 |
| rs2811388 | 0.908 | 1 | rs7374952 |
| rs2999081 | 0.908 | 1 | rs7374952 |
| rs2811518 | 1 | 1 | rs7374952 |
| rs11720239 | 1 | 1 | rs7374952 |
| rs745052 | 1 | 1 | rs745052 |
| rs7819002 | 0.918 | 1 | rs7460206 |
| rs750358 | 1 | 1 | rs750358 |
| rs17746918 | 1 | 1 | rs750358 |
| rs17399998 | 1 | 1 | rs7551796 |
| rs7605254 | 1 | 1 | rs7605254 |
| rs760645 | 1 | 1 | rs760645 |
| rs7621006 | 1 | 1 | rs7621006 |
| rs7702447 | 1 | 1 | rs7702447 |
| rs7739790 | 1 | 1 | rs7739790 |
| rs7760914 | 1 | 1 | rs7760914 |
| rs1452138 | 0.904 | 1 | rs7947323 |
| rs3824954 | 0.904 | 1 | rs7947323 |
| rs10876347 | 1 | 1 | rs7968748 |
| rs798766 | 1 | 1 | rs798741 |
| rs798766 | 1 | 1 | rs798755 |
| rs8139013 | 1 | 1 | rs8139013 |
| rs11864373 | 0.927 | 1 | rs904816 |
| rs9925768 | 0.927 | 1 | rs904816 |
| rs4377765 | 1 | 1 | rs9345095 |
| rs950590 | 1 | 1 | rs950590 |
| rs9604787 | 1 | 1 | rs9604787 |
| rs970694 | 1 | 1 | rs970694 |
| rs11864373 | 1 | 1 | rs9925768 |
| rs9925768 | 1 | 1 | rs9925768 |
