# Supplementary material for: TP53-based interaction analysis identifies cis-eQTL variants for TP53BP2, FBXO28, and FAM53A that associate with survival and treatment outcome in breast cancer
Source: Oncotarget. 2017 Feb 5;8(11):18381–98. doi: 10.18632/oncotarget.15110 (PMC5392336; doi:10.18632/oncotarget.15110)
Supplement: Supplementary file 5 [file oncotarget-08-18381-s005.docx]

**Supplementary Table 5.** Breast Cancer Association Consortium (BCAC) studies genotyped on the iCOGS chip and represented in this work.

| **Study Acronym** | **Study** | **Country** | **Cases^a^** | **Events^a^** | **Study design** |
| --- | --- | --- | --- | --- | --- |
| ABCFS | Australian Breast Cancer Family Study | Australia | 671 | 160 | Cancer registries in Victoria and New South Wales (1992-1999): all cases from Melbourne and Sydney diagnosed before age 40 plus a random sample of those diagnosed at ages 40-59. |
| ABCS | Amsterdam Breast Cancer Study | Netherlands | 443 | 60 | Breast cancer patients diagnosed before age 50 in 2003-2009 at the NKI-AVL; and (ABCS-F) All non-BRCA1/2 breast cancer cases from the family cancer clinic of the NKI-AVL tested in the period 1995-2009; all ages and diagnosed with breast cancer in 1965-2008. |
| BBCC | Bavarian Breast Cancer Cases and Controls | Germany | 248 | 16 | Consecutive, unselected cases with invasive breast cancer recruited at the University Breast Centre, Franconia in Northern Bavaria from 2002-2010. |
| ESTHER | ESTHER Breast Cancer Study | Germany | 228 | 41 | Statewide recruitment of breast cancer cases in all hospitals in Saarland/Germany in 2001-2003 |
| HEBCS | Helsinki Breast Cancer Study | Finland | 1451 | 199 | (1) Consecutive cases (883) from the Department of Oncology, Helsinki University Central Hospital 1997-8 and 2000, (2) Consecutive cases (986) from the Department of Surgery, Helsinki University Central Hospital 2001–2004, (3) Familial breast cancer patients (536) from the Helsinki University Central Hospital, Departments of Oncology and Clinical Genetics (1995-). |
| KARBAC | Karolinska Breast Cancer Study | Sweden | 409 | 106 | 1. Familial cases from Department of Clinical Genetics, Karolinska University Hospital, Stockholm. 2. Consecutive cases from Department of Oncology, Huddinge & Söder Hospital, Stockholm 1998-2000. |
| KBCP | Kuopio Breast Cancer Project | Finland | 410 | 154 | Women seen at Kuopio University Hospital between 1990-1995 because of a breast lump, mammographic abnormality, or other breast symptom and who were found to have breast Cancer. |
| kConFab/AOCS | Kathleen Cuningham Foundation Consortium for Research into Familial Breast Cancer / Australian Ovarian Cancer Study | Australia | 183 | 16 | Index (youngest affected) cases from BRCA1-and BRCA2-mutation-negative multiple-case breast and breast-ovarian families recruited though family cancer clinics from across Australia and New Zealand from 1998-present. |
| LMBC | Leuven Multidisciplinary Breast Centre | Belgium | 2074 | 67 | All patients diagnosed with breast cancer and seen in the Multidisciplinary Breast Centre in Leuven (Gashuisberg) since June 2007 plus retrospective collection of cases diagnosed since 2000. |
| MARIE | Mammary Carcinoma Risk Factor Investigation | Germany | 1635 | 244 | Incident cases diagnosed from 2001-2005 in the study region Hamburg in Northern Germany, and from 2002-2005 in the study region Rhein-Neckar-Karlsruhe in Southern Germany. |
| MCBCS | Mayo Clinic Breast Cancer Study | USA | 1198 | 100 | Incident cases residing in 6 states (MN, WI, IA, IL, ND, SD) seen at the Mayo Clinic in Rochester, MN from 2002-5 |
| OFBCR | Ontario Familial Breast Cancer Registry | Canada | 686 | 80 | Invasive cases aged 20-54 years identified from the Ontario Cancer Registry from 1996-1998. All those at high genetic risk were eligible; random samples of women not meeting these criteria were also asked to participate. |
| PBCS | NCI Polish Breast Cancer Study | Poland | 389 | 35 | Incident cases from 2000-2003 identified through a rapid identification system in participating hospitals covering ~ 90% of all eligible cases, and cancer registries in Warsaw and Łódź covering 100% of all eligible cases. |
| RBCS | Rotterdam Breast Cancer Study | Netherlands | 529 | 84 | Familial breast cancer patients selected from the clinical genetics centre at Erasmus Medical Centre between 1994-2005. |
| SASBAC | Singapore and Sweden Breast Cancer Study | Sweden | 1146 | 155 | Women diagnosed in Sweden aged 50-74 in 1993-1995. |
| SEARCH | Study of Epidemiology & Risk Factors in Cancer Heredity | UK | 6233 | 721 | Identified through the Eastern Cancer Registration and Information Centre: (I) prevalent Cases; diagnosed 1991-1996; under 55 years of age at diagnosis; recruited 1996-2002 (ii) incidence cases; diagnosed since 1996; under 70 years of age at diagnosis; recruited 1996-present. |
| SKKDKFZS^b^ | Städtisches Klinikum Karlsruhe Deutsches Krebsforschungszentrum Study | Germany | 123 | 21 | Women diagnosed with primary in situ or invasive breast cancer at the Städtisches Klinikum Karlsruhe from March 1993 to July 2005. Cases were 21-93 years of age. |
| ^a^ Counts indicate cases eligible for the current study (treatment and follow-up information available, follow-up time > recruitment latency).  ^b^ While SKKDKFZS is a BCAC study, this sample set was genotyped as part of the Triple Negative Breast Cancer Consortium. | | | | | |
