# Supplementary material for: TP53-based interaction analysis identifies cis-eQTL variants for TP53BP2, FBXO28, and FAM53A that associate with survival and treatment outcome in breast cancer
Source: Oncotarget. 2017 Feb 5;8(11):18381–98. doi: 10.18632/oncotarget.15110 (PMC5392336; doi:10.18632/oncotarget.15110)
Supplement: Supplementary file 6 [file oncotarget-08-18381-s006.docx]

**Supplementary Table 6.** TP53 immunohistochemical measurements and scoring methods for the BCAC studies with available TP53 data.

| **Study** | **Center** | **Measurement** | **Scoring** | **Positive scores (%)** | **Notes** |
| --- | --- | --- | --- | --- | --- |
|  |  |  |  |  |  |
| HEBCS | HEBCS | % positive nuclei | pos: > 20% positive nuclei | 22 % | a |
| KBCP | HEBCS | % positive nuclei | pos: > 20% positive nuclei | 18 % | b |
| MCBCS | HEBCS | % positive nuclei | pos: > 20% positive nuclei | 18 % | b |
| ESTHER | HEBCS | % positive nuclei | pos: > 20% positive nuclei | 15 % | b |
| kConFab/AOCS | HEBCS | % positive nuclei | pos: > 20% positive nuclei | 24 % | b |
| RBCS | HEBCS | % positive nuclei | pos: > 20% positive nuclei | 21 % | b |
| OFBCR | OFBCR | % positive nuclei and staining intensity | Allred's method; pos: score > 3 / 8 | 33 % | c |
| PBCS | PBCS | % positive nuclei (1-100) x intensity (1-3) | pos: score > 60 | 18 % | d |
| SEARCH | SEARCH | staining intensity and focality | neg: no staining, or weak focal staining; pos = strong diffuse staining | 14 % |  |
|  |  |  |  |  |  |
| 1. HEBCS TP53 staining and scoring has been performed previously using the same methodology as decribed in the current study. See Tommiska et al., 2005, for further details. 2. Staining and scoring performed centrally at HEBCS as described in the current study. 3. See Allred et al., 1998, for details on this scoring method 4. See Yang et al., 2007, for details on this scoring method | | | | | |

**Supplementary references pertaining to this table:**

Tommiska J, Eerola H, Heinonen M, Salonen L, Kaare M, Tallila J, Ristimäki A, von Smitten K, Aittomäki K, Heikkilä P, Blomqvist C, Nevanlinna H. Breast cancer patients with p53 Pro72 homozygous genotype have a poorer survival. Clin Cancer Res. 2005 Jul 15;11(14):5098-103.

Allred DC, Harvey JM, Berardo M, Clark GM. Prognostic and predictive factors in breast cancer by immunohistochemical analysis. Mod Pathol. 1998 Feb;11(2):155–168.

Yang XR, Pfeiffer RM, Garcia-Closas M, Rimm DL, Lissowska J, Brinton LA, Peplonska B, Hewitt SM, Cartun RW, Mandich D, Sasano H, Evans DB, Sutter TR, Sherman ME. Hormonal markers in breast cancer: coexpression, relationship with pathologic characteristics, and risk factor associations in a population-based study. Cancer Res. 2007 Nov 1;67(21):10608-17.
