# Supplementary material for: TP53-based interaction analysis identifies cis-eQTL variants for TP53BP2, FBXO28, and FAM53A that associate with survival and treatment outcome in breast cancer
Source: Oncotarget. 2017 Feb 5;8(11):18381–98. doi: 10.18632/oncotarget.15110 (PMC5392336; doi:10.18632/oncotarget.15110)
Supplement: Supplementary file 7 [file oncotarget-08-18381-s007.docx]

**Supplementary Table 7.** Candidate genes and corresponding siRNAs tested in the doxorubicin response experiment. Transfections were performed using Lipofectamine RNAiMAX (Thermo Fisher Scientific, Waltham, MA, USA). Cell viability was measured with the CellTiter-Glo luminescent cell viability assay (Promega, Madison, WI, USA).

| **Gene** | **siRNA id ^a^** |
| --- | --- |
|  |  |
| FAM53A | s45742, s45743, s45744 |
| SLBP | s15448, s15449, s15450 |
| TACC3 | s20469, s20470, s20471 |
| TMEM129 | s40915, s40916, s40917 |
| Negative control | Ambion Silencer Select Negative Control #1 |
|  |  |
| ^a^ All siRNAs originated from Thermo Fisher Scientific, Waltham, MA, USA. | |
